# Bacteria-Mediated Intracellular Radical Polymerizations

**DOI:** 10.1021/jacs.4c17257

**Published:** 2025-03-04

**Authors:** Eleonora Ornati, Jules Perrard, Tobias A. Hoffmann, Raissa Bonon, Nico Bruns

**Affiliations:** 1 Department of Chemistry and Centre for Synthetic Biology, Technical University of Darmstadt, Peter-Grünberg-Str. 4, 64287 Darmstadt, Germany; 2 Department of Pure and Applied Chemistry, University of Strathclyde, Thomas Graham House, 295 Cathedral Street, Glasgow G1 1XL, U.K.

## Abstract

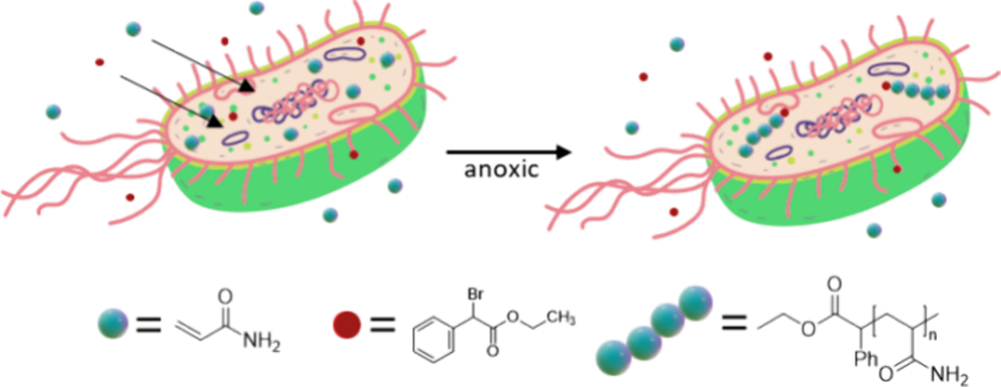

Intracellular radical polymerizations allow for the direct bioorthogonal synthesis of various synthetic polymers within living cells, thereby providing a pathway to polymer-modified cells or the fermentative production of polymers. Here, we show that *Escherichia coli* cells can initiate the polymerization of various acrylamide, acrylic, and methacrylic monomers through an atom transfer radical reaction triggered by the activity of naturally occurring biomolecules within the bacterial cells. Intracellular radical polymerizations were confirmed by nuclear magnetic resonance spectroscopy, gel permeation chromatography of polymers extracted from the cells, and fluorescence labeling of the polymer directly inside the cells. The effect of polymerization on cell behavior and the response of the cells to polymerization was investigated through fluorescence microscopy and flow cytometry techniques, as well as metabolic and membrane integrity assays. The polymer synthesis and resulting products are cell-compatible, as indicated by the high viability of the polymerized cells. *In cellulo* synthesis of synthetic polymers containing fluorescent dyes was also achieved. These results not only enhance our understanding of the untapped potential of bacterial cells as living catalysts for polymer production but also reveal intracellular polymerization based on atom transfer radical polymerization initiators as a bioorthogonal tool for cell engineering and synthetic biology.

## Introduction

Synthetic polymers play a crucial role in various domains such as everyday life, agriculture, and biomedical applications, establishing significant interactions with biological and living matter.^1,2^ However, the chemical synthesis of these macromolecules has been typically limited to environments outside of cells due to the harsh and often toxic conditions required.^1^ Polymerizations within and on cells offer a unique approach to modifying cellular environments. The functionalization of cell membranes and the formation of intracellular polymer networks can potentially enhance drug delivery and improve cell-based therapies, providing new tools to study and modify cellular functions.^3,4^ Additionally, polymerizations conducted directly within cells open up possibilities for developing advanced materials that mimic the dynamic nature of biological systems, offering a novel platform for the development of synthetic biological systems. Finally, intracellular polymerizations could be used to produce polymers by whole-cell biocatalysis in fermenters to contribute to the transition toward a bioeconomy. In recent years, initial strides have been made toward synthesizing polymers directly within the intracellular environment.^5−9^ Many of these studies focus on light-initiated radical polymerizations or require external inorganic catalysts.^10−16^ Intracellular polymerizations have been performed in eukaryotic cells, ranging from mammalian cells to fungi and plants, with few reports of such processes in prokaryotes like bacteria:^17^ polymers have been synthesized in genetically modified bacteria through reassignment of sense codons^40^ or the enzyme-mediated formation of hydrogels.^18^

*Escherichia coli*, a well-studied model organism, offers a robust platform for exploring the interplay between polymer chemistry and living matter. *E. coli*’s ease of genetic manipulation, rapid growth, and well-characterized physiology make it an ideal candidate for studying bioorthogonal reactions within a living system.^19,20^

**Figure 1:**
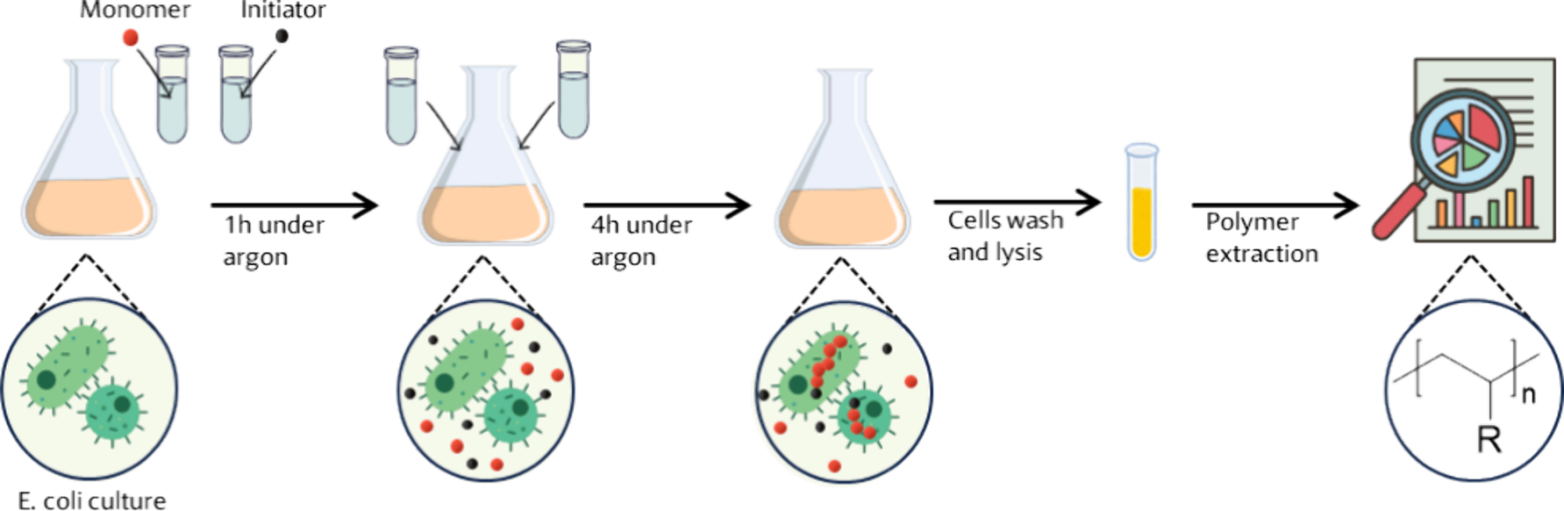
Schematic depiction of the experimental procedure for bacteria-mediated intracellular radical polymerizations. *E. coli* cells were grown in liquid culture, kept under argon for 1 h, and then mixed with degassed solutions of monomer and ATRP initiator. The reaction was stopped by introducing air, and the cells were washed to remove any extracellular residue. The polymer was then extracted and analyzed.

In this work, we investigate the potential of *E. coli* as host organisms for the intracellular synthesis of polymers. Our findings demonstrate that bacterial cells can initiate radical polymerizations using water-soluble atom transfer radical polymerization (ATRP) initiators, along with acrylamide, acrylate, and methacrylate monomers. This approach enables the bioorthogonal synthesis of polymers within the intracellular environment of bacterial cells [Figure 1]. We did not have to genetically modify the bacteria to express enzymes to catalyze the polymerizations. Natural catalysts present in the bacteria effectively initiate the polymerization, which then proceeds without compromising cell survival and proliferation. This opens new avenues for synthesizing polymers within bacterial systems, paving the way for innovative applications in synthetic biology and biotechnology.

## Result and Discussion

### *E. coli* Extract Catalyzes Polymerization Reactions

A possibility of interfacing polymer chemistry with biological systems is to use proteins as biocatalysts for polymerization reactions. Notably, metalloenzymes and metalloproteins have successfully mediated ATRP and initiated reversible addition–fragmentation chain transfer (RAFT) polymerizations, producing various polymers.^21−23^ Particularly, ATRP-like reactions have been catalyzed by heme-containing proteins, such as hemoglobin,^24−26^ myoglobin,^27^ and horseradish peroxidase,^28−30^ in both aqueous solutions and complex biological fluids like blood samples.^25,31^

**Figure 2:**
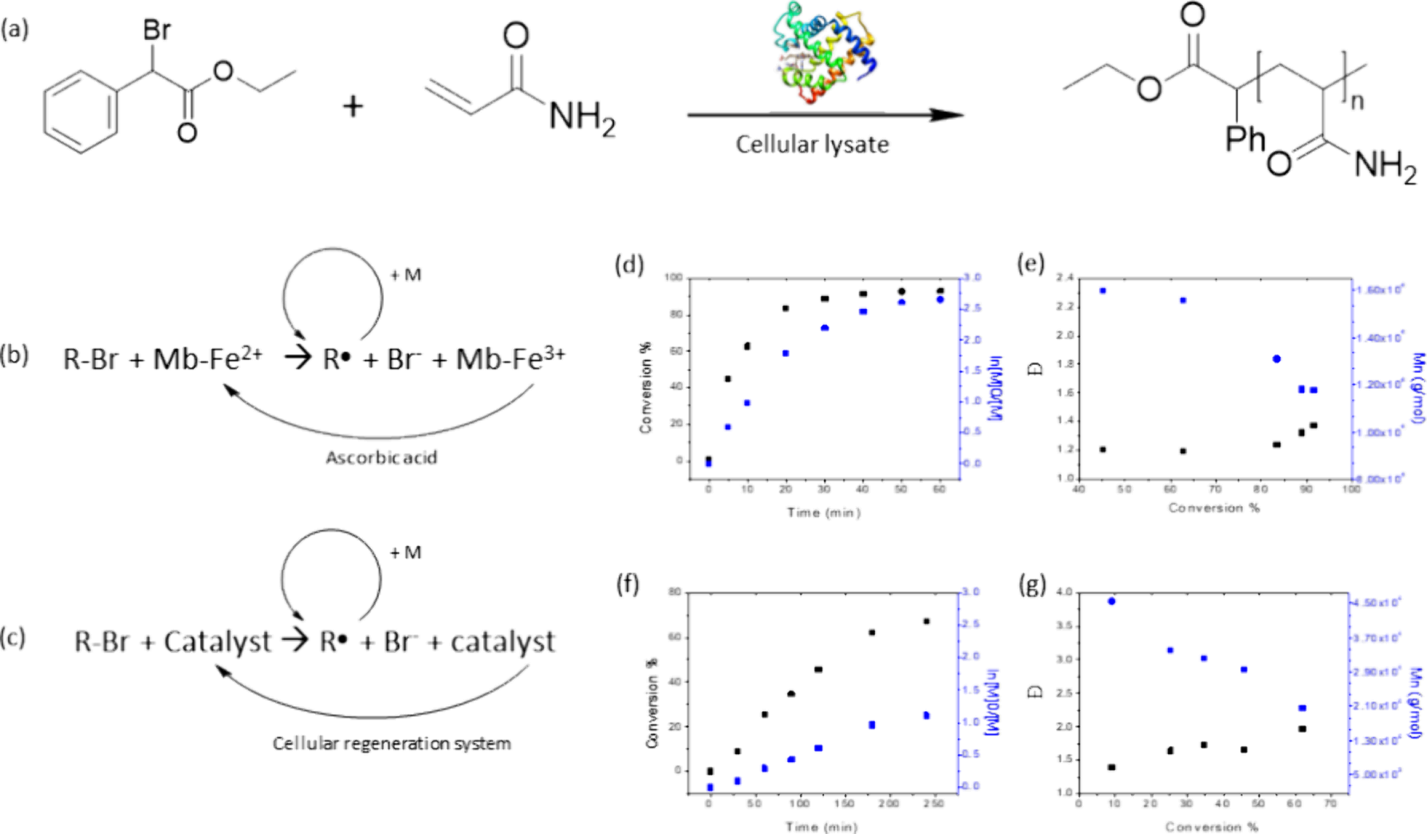
Biocatalytic polymerizations in PBS and in cellular lysate, catalyzed by myoglobin and by an endogenous catalyst. (a) Reaction scheme of the polymerization in cellular lysate from *E. coli* BL21(DE3). (b) Proposed reaction mechanisms for the myoglobin-catalyzed polymerization in PBS. (c) Proposed mechanism for the polymerization with endogenous catalysts in the cell lysate (and within cells). (d–g) Kinetics of the polymerization of AAm in cellular lysate from *E. coli* BL21(DE3) without the addition of an external catalyst. (d, e) 1.55 M AAm, 0.31 × 10^–3^ M EBPA (50:1) in dense cellular lysate; (f, g) 0.6 M AAm, 0.31 × 10^–3^ M EBPA (20:1) with 1.5 × 10^–2^ M NaAsc in 1.2-fold diluted cellular lysate in PBS.

To first understand whether an enzyme-catalyzed polymerization in the overcrowded inner space of bacteria could be possible, polymerizations were carried out in lysate obtained from *E. coli* cells that were cultured in a liquid medium, harvested, and then lysed by ultrasonication. The obtained cellular lysate mimics the composition and concentration of components inside the cells, and it was used directly as a reaction medium for the polymerizations. Based on previous studies on protein-catalyzed polymerization in aqueous media,^24,25,31^ the heme-containing protein myoglobin was chosen as a model catalyst to investigate ATRP-like reactions in the cellular lysate. Myoglobin efficiently triggered the polymerization by debromination of ATRP initiators in phosphate-buffered saline (PBS) and in cellular lysate, using sodium ascorbate (NaAsc) to reduce the iron to its +2 oxidation state [Figure 2a,b, and Tables S1 and S2]. Initially, reactions involving myoglobin, acrylamide (AAm), and *N*-acryloyl morpholine (NAM) as model monomers and ethyl α-bromo phenylacetate (EBPA) as case of study initiator were carried out. Despite the overcrowded conditions of the lysate and the presence of many molecules that could prevent the reaction from proceeding, like reducing agents and radical scavengers, it proved to be a suitable environment for radical polymerizations. Thus, further studies were carried out to understand the optimal reaction conditions. The effect of temperature was first explored to optimize the turnover and efficiency of the polymerization in cellular lysate [Table S1]. For both monomers, higher conversion and better dispersity were achieved at 37 °C than at room temperature. Therefore, the temperature of 37 °C was chosen for all further reactions, including polymerization in living bacteria cells unless otherwise stated.

The contributions of myoglobin and reducing agents, particularly NaAsc, were tested [Table S2]. We first explored the possibility of performing the polymerizations in buffer and lysate without adding the reducing agent. As expected, the reaction did not work in PBS, where commercial myoglobin in the Fe^3+^ state was used. However, the reaction proceeded well in the cellular lysate. No polymer was obtained for the reaction in PBS without the protein and reducing agents, while the presence of the reducing agent NaAsc without myoglobin was enough to reach a conversion of about 10%. In the cellular lysate, polymers were obtained with and without added myoglobin. The presence of commercial myoglobin did not result in any significant changes in the dispersity of the final polymer. The presence of reducing agents resulted in a low yield of around 25% when myoglobin was not added to the mix and a smaller molecular weight of the final polymer, independent of the presence of the heme-containing protein. Eliminating reducing agents and the commercial myoglobin yielded polymers with a higher molecular weight but similar dispersity to the reaction performed with all of the components. The presence of NaAsc resulted in a longer reaction time to achieve high conversion. Without the reducing agent, a final conversion of more than 80% could be achieved in less than 1 h. In contrast, for the same reaction conditions but with NaAsc, a conversion of around 80% was reached in 4 h, with a conversion of 35% in 1 h. Based on the data collected, NaAsc was able to trigger the polymerization reaction, probably due to its easy oxidation. At the same time, it slows the polymerization, given its known antioxidant activity, resulting in longer reaction times. The presence of the monomer alone, without an initiator, resulted in a conversion of only 3% in cellular lysate, confirming the atom-transfer-based initiation mechanisms and the need for an ATRP initiator.

Finally, the effect of a recombinantly overexpressed myoglobin was tested (Table S2). The reactions were conducted in cellular lysate from cells that had been transformed with an exogenous wild-type (WT) sperm whale myoglobin gene cloned in a pET expression vector. The overexpression of WT myoglobin did not change the conversion, molecular weight, or dispersity of the synthesized polymers, allowing us to conclude that a recombinantly expressed protein catalyst within the bacteria has no major effects on the reaction.

In conclusion, polymers can be synthesized in the cellular lysate. However, myoglobin, either extrinsically added or intrinsically produced, does not provide better results in terms of dispersity and molecular weight. Without the heme-containing protein, the polymerization yields polymers with a relatively low dispersity down to *Đ* = 1.4. The mere presence of monomer and initiator in cellular lysate under anaerobic conditions is sufficient for the polymerization, making the process easier to perform and revealing how *E. coli* cells already contain the necessary catalysts to trigger the polymerization [Figure 2c].

#### Polymerization Kinetics in Cellular Lysate

The kinetics of the EBPA-initiated polymerization of AAm in the cellular lysate were studied [Figure 2d–g]. The reaction proceeded very fast, reaching a conversion of around 60% in 10 min. The viscosity, already high because of the dense cellular lysate, increases drastically during the reaction because of the formation of polymer chains. Linear conversion and first-order kinetics were observed in the first 20 min. Then, the reaction slowly reached a plateau with a final monomer conversion of 90% in 1 h [Figure 2d]. The dispersity increased during the reaction, starting from 1.2 and reaching 1.4 at the highest conversion, while the number-average molecular weight *M*_*n*_ decreased [Figure 2e]. The decrease in *M*_*n*_ with conversion suggests that the initiation reaction overlaid the propagation reaction and that throughout the polymerization, new chains formed, which did then not reach the high molecular weights of the chains that formed at the beginning of the reaction due to the consumption of the monomer. This is in accordance with a suspected protein-catalyzed reaction, where the debromination of the ATRP initiator (and thereby the initiation of polymerization) by a protein is slow and proceeds over a certain period of time.

To prevent the viscosity from increasing during the reaction, the kinetic experiment was repeated in a diluted cellular lysate, and the amount of acrylamide was reduced by a factor of 2.5 to yield lower molecular weight polymers. Moreover, NaAsc was added to slow the reaction. The kinetics of this reaction were also of first order, and a plateau in conversion was reached after 3 h [Figure 2f]. The final conversion was 70%, i.e., around 20% less than in the previous reaction. Molecular weight and dispersity followed the same trend as in the reaction in undiluted lysate (Figure 2g). The dispersity of the obtained polymer was higher, and the molecular weight was smaller, likely because of the lower monomer concentration in the reaction.

The preparation of the cellular lysate and its final concentration are crucial parameters that can result in significant changes (Figure 2f,g). Diluted lysate was found to result in lower conversion and higher dispersity, most likely due to the lower concentration of catalysts in the mix.

#### Screening of Monomers and Initiators

In order to establish suitable reaction conditions for intracellular polymerizations, several monomer and initiator combinations were tested. A restrictive condition for polymerization in aqueous media is the solubility of the monomer and initiator. Because of that, only water-soluble monomers and, at least slightly, water-soluble initiators were chosen. The ATRP initiator EBPA is known to be one of the most active ones in conventional ATRP reactions, which is essential to ensure high initiation efficiency.^32^ For these reasons, EBPA was tested against several different monomers, resulting in successful polymerizations in many cases [Table S3]. The ATRP initiators 2-hydroxyethyl-2-bromoisobutyrate (HEBIB), α-bromo phenylacetic acid (BPAA), *N*-isopropyl-2-bromopropionamide (NIPBPA), and methyl α-bromo phenylacetate (MBPA) were also tested for the polymerization of various monomers and successfully polymerized monomers such as AAm, *N*-isopropylacrylamide (NIPAm), NAM, and 2-hydroxypropyl methacrylate (HPMA). Overall, almost all the initiators and monomers tested provided good polymerization efficiency in terms of conversion and short reaction time, while only a few of them, including 2-bromopropionitrile (BPN), did not work under our conditions [Table S3]. The initiator NIPBPA resulted in a slightly lower yield than the other initiators and was, therefore, not selected for further examination in cells [Table S3, Figures S1 and S2].

The cytotoxicity of monomers and initiators was another important parameter to study before polymerizations were carried out in living cells. To estimate the harmfulness of the molecules and rank them on a scale of toxicity, the half-maximal inhibitory concentration (IC_50_) was determined for a single compound after 6 h of incubation at 37 °C [Figure S3]. The initiators showed higher toxicity compared with almost all monomers tested, with the worst being EBPA (0.16 mM) and the best being HEBIB (4.33 mM). Monomers such as AA (166.26 mM), NIPAm (92.50 mM), and NAM (58.29 mM) showed good biocompatibility. The monomer *N*-(2-hydroxypropyl)methacrylamide (HPMAm) showed one of the lowest cytotoxicity (157.67 mM), but no polymer was obtained with this monomer in any condition tested [Table S3]. In contrast, the initiator methyl α-bromo phenylacetate (MBPA) showed good polymerization efficiency but resulted in very low viability of the cells after treatment [Figure S4]. For these reasons, both of them were excluded from further studies.

Based on the data collected, AA, NAM, NIPAm, HPMA, EBPA, HEBIB, and BPAA were selected as possible candidates for successful polymerization in living cells [Figure 1].

#### Polymerization in Cells

For polymerizations in living cells, *E. coli* BL21(DE3), carrying a vector for antibiotic resistance, was grown in Luria–Bertani broth (LB) for 5 h to an OD_600_ between 1.8 and 2. The cells were used directly in the growth medium unless otherwise stated. Cells were kept under argon for at least 1 h in a sealed bottle before the polymerization to ensure the removal of oxygen from the culture. *E. coli* is a metabolically versatile bacterium that can grow in both aerobic and anaerobic environments by shifting its metabolism from a respiratory chain that uses oxygen as an electron acceptor to a respiratory chain that uses alternative electron acceptors. *E. coli* can thus survive and be metabolically active in oxygen-depleted environments.^33^

**Figure 3:**
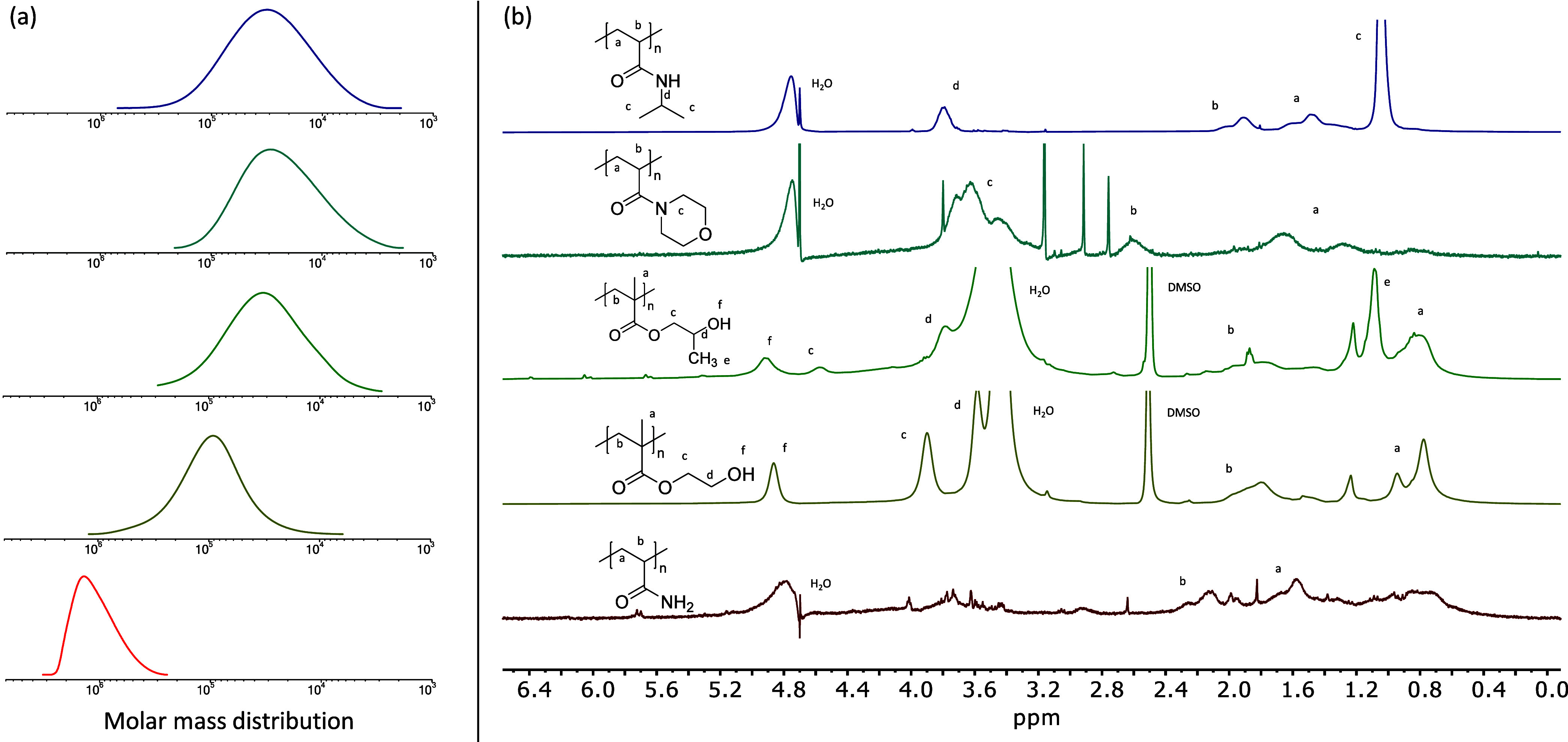
Intracellular polymerizations in *E. coli* BL21(DE3). (a) GPC chromatograms of polymers extracted from cells. Purple: PNIPAm, blue: PNAM, green: PHPMA, yellow: PHEMA, red: PAAm. (b) ^1^H NMR spectra of polymers extracted from cells. Impurities and unlabeled peaks are due to not identified substances extracted from the cells together with the polymer of interest. In contrast to the other polymers, PAAm was measured in diluted cell lysate, thereby giving rise to more peaks from components of the lysate than in the spectra of the other polymers. PAAm, PNAM, and PNIPAm NMR spectra in D_2_O; PHEMA and PHPMA NMR spectra in DMSO-*d*_6_.

Initiator and monomer solutions were degassed separately before being injected into the bacteria bottle (Figure 1). Considering the cytotoxicity of the chemicals needed for the polymerization, an initiator concentration of 1 mM and a monomer concentration of 50 or 20 mM were chosen unless otherwise noted. A final concentration of 50 mM was used for monomers, resulting in water-soluble polymers (AAm, NIPAm, and NAM) and 20 mM for monomers that give water-insoluble polymers (HEMA and HPMA). The concentrations were first tested in cellular lysate with NIPAM, and PNIPAm was obtained under these conditions (Figure S5). Then, different monomers and initiators were tested in the cells. The best initiator in terms of polymerization efficiency—EBPA—was used to polymerize AAm, NIPAm, and HEMA. The intracellular polymerization was confirmed by NMR spectroscopy and gel permeation chromatography (GPC) [Figure 3, Table S4]. To this end, cells were accurately washed in PBS after the reaction and lysed. The polymers were extracted from the cellular lysate according to their physio-chemical properties. PNIPAm and PHEMA could be successfully extracted from the cells. The polymers had a dispersity of 1.57 and 1.55, and a *M*_*n*_ of 7.73 × 10^4^ and 1.06 × 10^4^ g mol^–1^, respectively. In contrast, polyacrylamide was very difficult to extract from cell lysate because of its high solubility in water. It was not possible to separate the low-concentrated polymer from the highly concentrated protein solution obtained after cell lysis. We could only isolate PAAm when a high monomer concentration of 150 mM was used, which led to high molecular weight polymers of *M*_*n*_ > 10^6^ g mol^–1^.

The initiator HEBIB also performed well and led to the successful polymerization of NIPAm, NAM, HPMA, and HEMA in cells [Table S4]. In contrast, no polymers formed with BPAA as an initiator when NIPAm or NAM were polymerized in the cells [Figure S6]. The nature of the initiator might explain this. BPAA has a p*K*_a_ of about 2.21^34^ and is, therefore, deprotonated at pH 7.4. In this state, it might be repelled from the surface of the bacteria, which is negatively charged.^35^

Using HEBIB as initiator, the polymerization in cells resulted in lower dispersity and lower molecular weight compared to the reaction conducted in the cellular lysate (for NAM, *Đ* = 1.73 and *M*_*n*_ = 1.96 × 10^4^ g mol^–1^ in cells compared to *Đ* = 2.89 and *M*_*n*_ = 1.28 × 10^5^ g mol^–1^ in cellular lysate) [Table S5]. This might be due to the higher catalyst concentration in the cell’s inner space or to a slower diffusion and lower mobility of the growing chains resulting from the high density of the bacterial cytoplasm, which might prevent termination reactions from happening.

To confirm the necessity of an ATRP initiator, control experiments were conducted in which the cells were incubated only with NIPAm, and the initiator solution was replaced with pure DMSO. No extractable polymer was obtained, which confirms the necessity of an ATRP initiator [Figure S7].

Parallel experiments in aerobic and anaerobic conditions show that oxygen is detrimental to the polymerization. After the reaction of NIPAm with EBPA in the presence of air, no polymer could be extracted from the cells [Figure S7]. Oxygen might not prevent the reaction from happening in absolute terms. Still, it could lead to very low yields and short polymer chains that were not detected in the NMR and GPC measurements.

**Figure 4:**
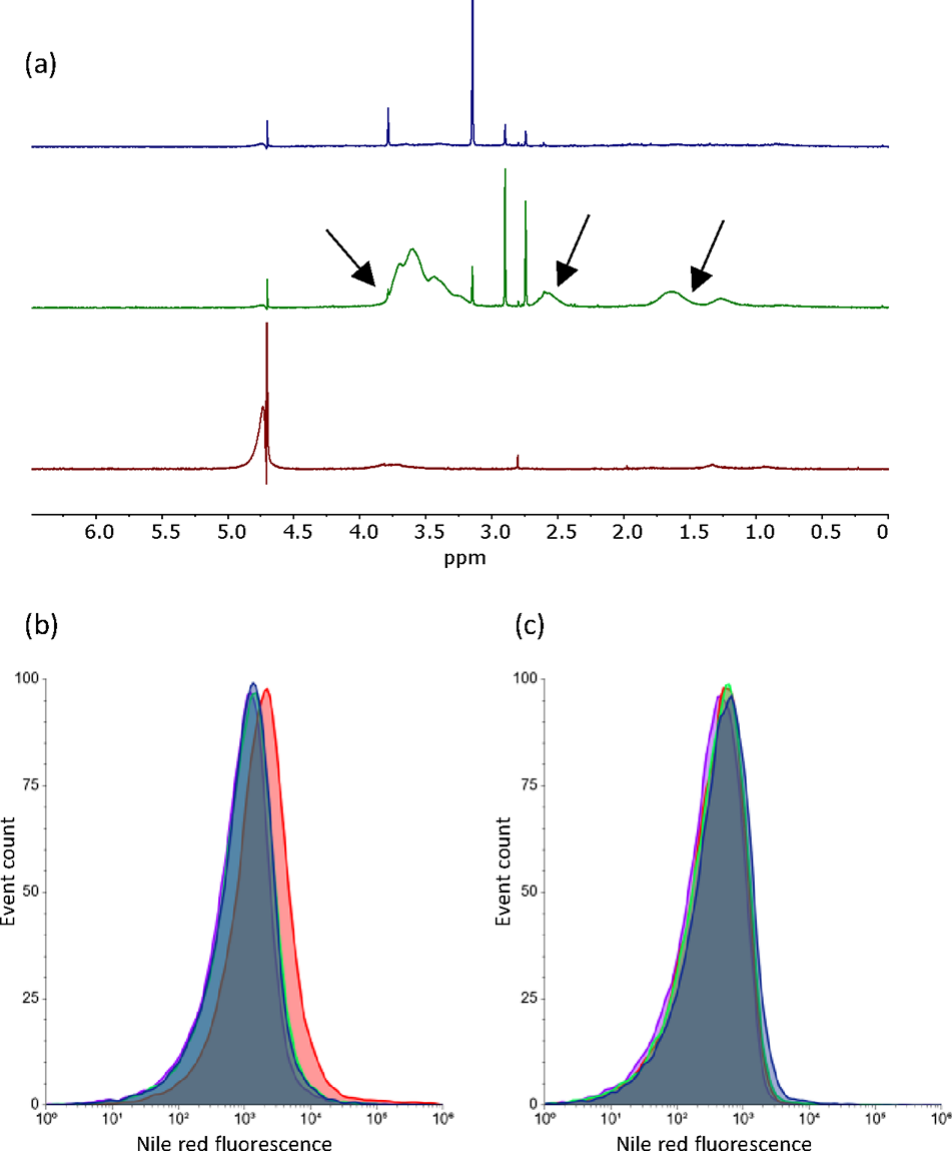
Evidence that the polymers synthesized by *E. coli* were located inside the cells. (a) Comparison of ^1^H NMR spectra of reactions of NAM with HEBIB as the initiator. Blue: extract of *E. coli* cells that were added to LB medium in which a possible polymerization had been carried out before; green: extract of *E. coli* cells that were polymerized while being suspended in PBS instead of LB; red: final wash of the polymerized cells. Arrows indicate the typical peaks of PNAM. (b, c) Flow cytometry analysis of *E. coli* cells in which intracellular PHEMA was stained with Nile Red (ex: 561 nm, em: 585 nm) with the addition of glycerol (b) and without glycerol (c). The use of glycerol during staining helps the dye pass the cell membrane, allowing it to stain the intracellularly located polymer. Red: HEMA+ HEBIB+; blue HEMA+ HEBIB–; green: HEMA– HEBIB+; purple: HEMA– HEBIB–.

Several control experiments were then performed to confirm that the polymerization happened inside the cells [Figure 4]. To rule out the possibility that the polymer formed in the LB medium used to suspend the cells and would then stick to the cells, cells were removed by centrifugation from the medium in which they had grown. Then, NAM polymerization was carried out in this medium. After 4 h of reaction, cells were added to the mixture, which was then incubated for 1 h in aerobic conditions. The cells were washed and lysed, but no detectable polymer was extracted from the cells [Figure 4a, blue spectrum]. Thus, the polymers observed in the other experiments had formed in the cells and did not result from spontaneous polymerizations in the LB medium.

To further test that the polymerization occurred within the cells and not in the LB medium, cells were polymerized in PBS. To this end, the cells were washed to remove any LB medium traces. Then, the polymerization of NAM was carried out as described above. Thereafter, the cells were washed three times with 50 mL of PBS and harvested. The polymer was extracted from the cell pellet and analyzed by NMR spectroscopy, confirming that polymerization had occurred [Figure 4a, green spectrum]. Moreover, 10 mL from the last wash in PBS of the polymerized cells were dried and resuspended in 1 mL of D_2_O for analysis by NMR spectroscopy. No polymer was found in such a sample, confirming the absence of polymer outside the cells [Figure 4a, red spectrum].

To prove that the polymers formed within the cells and not on their surface, PHEMA polymers were stained with Nile Red. The dye is known to stain lipids and hydrophobic microplastics, including many polymers.^36,37^ Polymerized cells that were treated with HEMA and HEBIB, and control cells were washed in PBS and resuspended in a Nile Red staining solution. In flow cytometry experiments, the mean fluorescence intensity of polymerized cells was 2-fold higher than the fluorescence of control cells when glycerol was added to the staining solution, which makes the membrane permeable for the dye, confirming the intracellular localization of the polymer [Figure 4b]. Without glycerol, all cells showed a similar fluorescence because of the poor membrane permeability of Nile Red (Figure 4c]. Moreover, Nile Red fluorescence was recorded for polymerized and control cells before and after sonication [Figure S8]. Before the disintegration of the cell wall, control and reacted cells fluoresced with similar intensity. However, the lysate obtained from the polymerized cells showed higher fluorescence intensity than the lysate of the control cells, most likely due to the release of PHEMA into the solution and its subsequent staining with Nile Red, which indicates that polymer resided within the cells and not on their surface.

#### Polymerizations with Fluorescent Monomers

To find an easier way to detect the formation of polymers inside the cells, copolymers of acrylamide and fluorescein-*O*-methacrylate (FOM) were synthesized in cellular lysate using EBPA as initiator. A control reaction was performed by repeating the same experiment without the initiator. After the reactions were concluded, the reaction mixtures were analyzed by aqueous GPC and sodium dodecyl sulfate-polyacrylamide gel electrophoresis (SDS-PAGE) [Figures S9 and S10]. The GPC chromatogram of the polymerization shows a polymer signal in the refractive index (RI) and UV–vis channels, while no signal was found for the control reaction. Moreover, the SDS-PAGE separated the copolymer from the unreacted fluorescent monomer. In addition, the copolymer was purified from the unreacted monomers by size exclusion chromatography, and the obtained polymer solution was strongly fluorescent [Figure S11]. All of these results indicate that a fluorescent P(AAm-*co*-FOM) copolymer formed in the lysate polymerization.

A fluorescent copolymer of NIPAm and FOM, using HEBIB as the initiator, was also synthesized in the cellular lysate. The synthesis of the copolymer was confirmed by SDS-PAGE and by precipitating it at 50 °C and redissolving the thus purified polymer in ultrapure water at room temperature, which resulted in a fluorescent solution [Figures S12 and S13].

**Figure 5:**
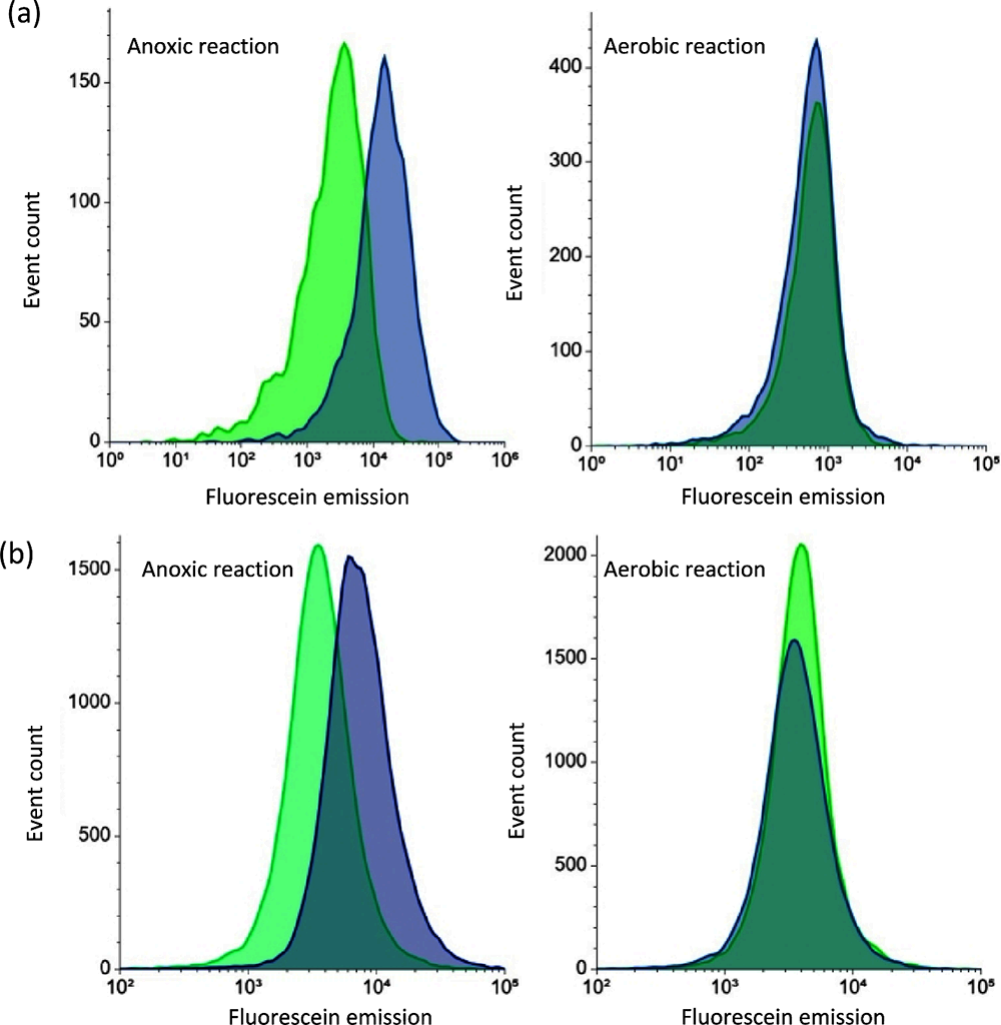
Intracellular copolymerization of a fluorescent monomer in *E. coli*. Flow cytometry analysis (ex: 488 nm/em: 520 nm) of (a) cells treated with 50 mM AAm and 0.02 mM FOM with (blue) and without 1 mM EBPA as initiator (green) in anoxic (left) and open-air (right) conditions. (b) Cells treated with 50 mM NIPAm and 0.02 mM FOM with (blue) and without 1 mM HEBIB as initiator (green) in anoxic (left) and open-air (right) conditions. The fluorescence intensity of the cells increased only when all of the conditions for intracellular polymerization were fulfilled.

The experiments were then adapted to living cells. To this end, *E. coli* cells were exposed to 50 mM AAm or NIPAM, 0.02 mM FOM, and 1 mM initiator (EBPA for AAm and HEBIB for NiPAm) and were allowed to react in anaerobic conditions for 4 h. A control experiment was conducted in parallel, where cells were fed only with the two monomers. After the reaction, cells were washed and analyzed by flow cytometry. The polymerized cells showed a higher fluorescence intensity than the control cells [Figure 5]. The same reaction in aerobic conditions, where no or only very small amounts of polymer formed, did not lead to an increased fluorescence of the cells, indicating that polymer formation caused the observed increase in fluorescence in anaerobic conditions. After the cells had been lysed, the fluorescence of supernatants from reacted and control cells confirmed the higher fluorescence intensity of the reacted cells. The supernatant obtained from the reacted cells was 2.3 times more fluorescent than the control [Figure S14a,c]. Finally, the polymers were purified by SEC. The fluorescence of the product obtained from the polymerized cells was markedly stronger than the fluorescence of the product from the control reaction [Figure S14b,d]. These results suggest that the reacted cells, treated with both monomers and the initiator under anaerobic conditions, accumulated the dye upon polymerization, which led to the higher fluorescence recorded.

#### Effect of Intracellular Polymerizations on Cells

**Figure 6:**
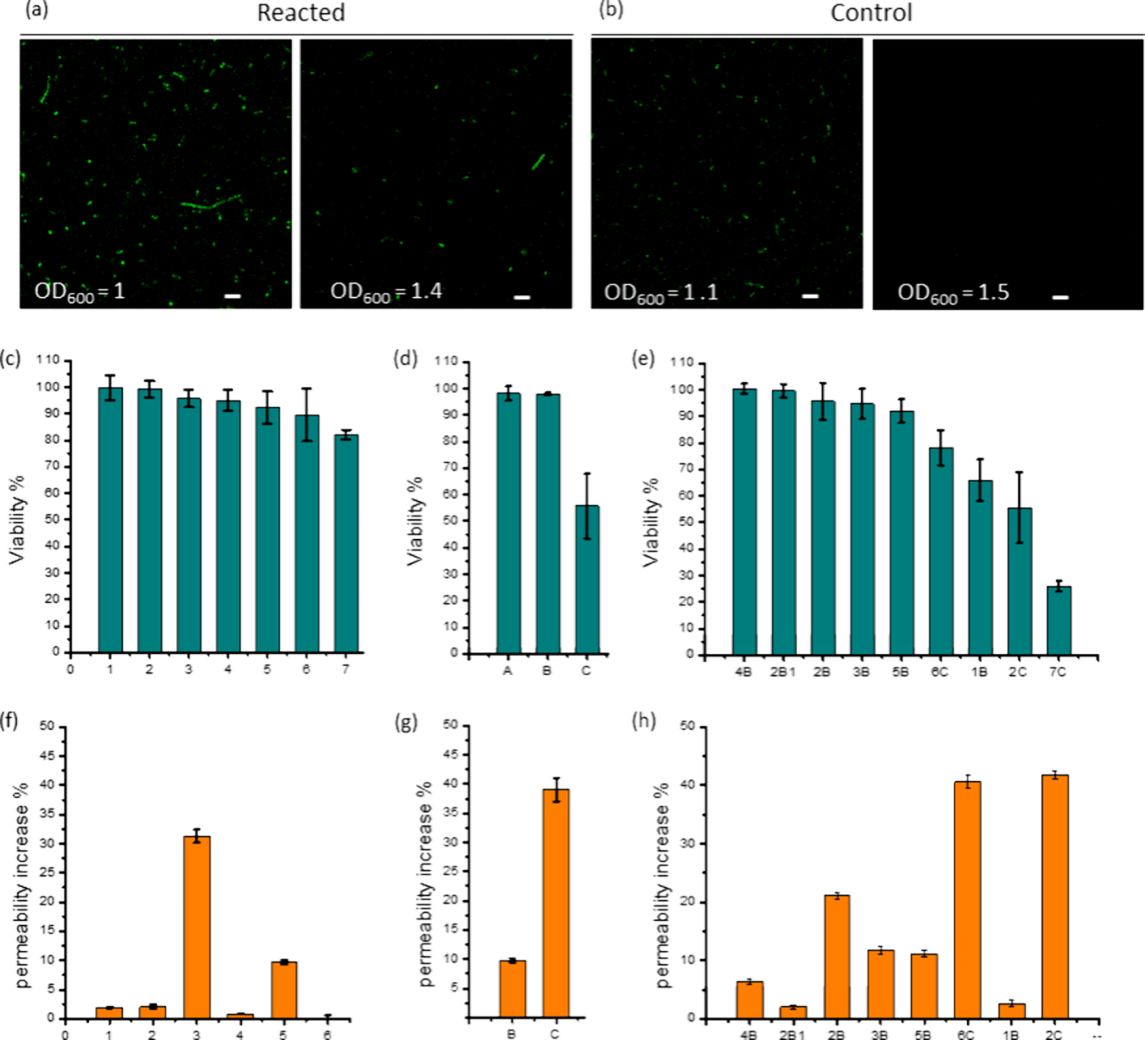
Effect of intracellular polymerizations on *E. coli* BL21(DE3) cells. (a) Confocal microscopy images (ex: 488 nm/em: 500–600 nm) of polymerized and of control cells directly after 4 h of reaction (reacted cells: 50 mM NIPAm, 0.02 mM FOM, 1 mM HEBIB; control cells: 50 mM NIPAm, 0.02 mM FOM), and after the cells were washed, diluted, and then allowed to continue to grow in fresh LB for 3 h. Reacted cells were still fluorescent after 3 h of growth, while the nonpolymerized control cells did not retain their fluorescence. Scale bars = 5 μm. (c–e) Cell viability as measured in CFU experiments and (f–h) membrane permeability as measured by PI fluorescence intensity, after treatment of the cells with monomers. (1) 20 mM HPMA; (2) 50 mM NIPAm; (3) 50 mM NAM; (4) 50 mM NIPAm + 0.02 mM FOM; (5) 20 mM HEMA; (6) 50 mM AAm + 0.02 mM FOM; (7) 150 mM AAm, initiators. (A) 1 mM BPAA; (B) 1 mM HEBIB; (C) 1 mM EBPA), or a combination of the two, which leads to the intracellular formation of polymers. All reactions were carried out under anaerobic conditions at 37 °C, except for 2B–28 °C, i.e., the polymerization of NIPAm below the LCST of the polymer, which was conducted at 28 °C. Mean values of *n* = 3 measurements ± SD are reported.

After demonstrating that *E. coli* can autonomously polymerize acrylamide and (meth)acrylate monomers in the presence of an ATRP initiator, it was interesting to investigate the cells’ response to the intracellular polymerizations. Therefore, the effect of the intracellularly located polymers on the subsequent growth of the cells was investigated. Because of the biocompatibility of HEBIB, NIPAm, FOM, and the resulting polymer, the system was chosen as a case study. Polymerized cells and control cells were first washed in PBS, then diluted, and allowed to grow in fresh LB media for 3 h. The growth was followed by measuring the OD_600_. There was no difference in duplication time between polymer-containing cells and control cells, with the cell density for both cell types increasing at similar rates [Table S6]. After 3 h of growth, polymerized cells were still fluorescent, while no cells were fluorescent in the control experiments, as shown in confocal microscopy [Figure 6a,b]. The residual high fluorescence of some of the polymerized cells could come from those cells that showed higher fluorescence after polymerization, or it might be due to a nonhomogeneous partition of the cytoplasmatic content between daughter cells.

Furthermore, we tested the ability of the polymerized cells to proliferate by determining the colony-forming units (CFU) of cells after treatment with various monomers, initiators, or a mixture of the two, the latter resulting in polymers under anaerobic conditions. Treated and untreated cells underwent the same process and were subsequently diluted and plated on LB agar, and the colonies were grown. In general, cells showed a high tolerance to most monomers and initiators, and cell viability was not reduced upon polymerization [Figure 6c–e]. However, the most toxic compound was EBPA, which gave results comparable to those of its cytotoxicity rank. The colonies obtained after treatment with EBPA appeared to be very irregular in shape and dimension, with slower growth than the untreated ones, a clear sign of cytotoxicity of the compound [Figure S15]. Moreover, the toxicity appeared stronger if EBPA was added to the cells before establishing an anoxic environment (Figure S16]. When EBPA was added after degassing in an anoxic condition, it resulted in half of the cells being dead or not able to duplicate. Based on CFU counting, treatment of the cells with 1 mM EBPA for 4 h resulted in 55% cell survival. In contrast, the other two initiators tested had almost no effect on cell viability, with a survival of 98% for both HEBIB and BPAA at a final concentration of 1 mM. All monomers resulted in a survival above 80% at any concentrations tested, while adding 0.02 mM FOM resulted in a slight decrease in the viability of the cells. Upon polymerization, PHPMA reduced the cell viability to 65% after 2 h of reaction. Polyacrylamide obtained at a high monomer concentration of 150 mM was the worst in terms of cell survival (26% viable cells), showing an increased number of cells with damaged membranes and a decreasing number of metabolically active cells as the reaction progressed [Figure S17]. All other polymers did not considerably affect the cell viability more than the single components alone, with a survival rate of around or above 90% when HEBIB was used as the initiator.

Because of the temperature-responsiveness of PNIPAm [Figure S18], the survival of the cells upon the polymerization of NIPAm above and below its lower critical solution temperature (LCST) was also analyzed. NIPAm polymerizations were carried out at 28 °C, where the polymer is soluble, and 37 °C, where the growing polymer chains precipitate. When polymers were formed at 28 °C, the survival of the cells was around 100%. When the polymers were synthesized at 37 °C, cell viability was around 95%, i.e., slightly lower [Figure 6e], most likely because the precipitation polymerization stressed the cells more than the synthesis of soluble polymers.

Finally, the membrane integrity of the cells was investigated with the help of the DNA-binding dye propidium iodide (PI), which does not permeate through the membrane if it is intact. In contrast, if pores form or the membrane becomes permeabilized, the dye can enter the cells and intercalate to the DNA, resulting in high fluorescence intensity.^38,39^ In general, the integrity of the membrane was well preserved for many conditions tested [Figure 6f–h]. Cells treated with EBPA or NAM showed the highest membrane permeability to PI, but the damage to the membrane decreased slightly when NAM was polymerized, while the presence of EBPA as an initiator caused the membrane to be more permeable even after polymerization. Surprisingly, PHPMA, which resulted in one of the lowest cell survival rates after polymerization (c.f. Figure 6e entry 1B), did not significantly decrease the membrane integrity compared to the control or other treatments tested, suggesting a bacteriostatic mechanism that does not affect the integrity of the membrane.

## Conclusions

In conclusion, we demonstrate the ability of *E. coli* to drive the intracellular polymerization of different acrylamide, acrylate, and methacrylate monomers through an ATRP-like initiation without the need to express enzymatic polymerization catalysts recombinantly or to add polymerization catalysts externally. Polymers were successfully synthesized directly inside the cells, and many demonstrated high biocompatibility without interfering with cells’ behavior and replication. The copolymerization of a fluorescent monomer with nonfluorescent monomers gave the advantage of easy and fast monitoring of the success of the reaction. The copolymerization of the fluorescent monomer also produced long-lasting fluorescence of the reacted cells during bacterial growth. Overall, this work is a step toward developing semiartificial cells, enabling the synthesis of synthetic macromolecules within living cells without deleterious problems for the microorganisms. The production of polymers directly inside the cells is a bioorthogonal process that could lead to novel engineered living materials or be used to produce synthetic polymers by whole-cell biocatalysis.

## Supporting Information Available

Detailed experimental procedures, GPC data and monomer conversion, mass spectrum, NMR spectra, inhibition curves, cytotoxicity data, fluorescence spectra, SDS-PAGE results, photos of reaction mixtures, OD_600_ measurements, confocal microscopy images, and cloud point measurements (PDF)
