# Supplementary material for: Bacteria-Mediated Intracellular Radical Polymerizations
Source: J Am Chem Soc. 2025 Mar 4;147(11):9496–504. doi: 10.1021/jacs.4c17257 (PMC11926860; doi:10.1021/jacs.4c17257)
Supplement: Supplementary file 1 — ja4c17257_si_001.pdf [file ja4c17257_si_001.pdf]

SUPPORTING INFORMATION

Bacteria-mediated intracellular radical polymerizations

Eleonora Ornati,[1,2] Jules Perrard,[1] Tobias A. Hoffmann,[1] Raissa Bonon,[1] Nico Bruns[1,2]*

[1]Department of Chemistry and Centre for Synthetic Biology, Technical University of Darmstadt, Peter-Grünberg-Str. 4, 64287 Darmstadt, Germany

[2]Department of Pure and Applied Chemistry, University of Strathclyde, Thomas Graham House, 295 Cathedral Street, Glasgow G1 1XL, UK

*nico.bruns@tu-darmstadt.de

Materials and Methods

Materials

Monomers and initiators were purchased on Sigma-Aldrich at the highest purity available and used directly without further purifications if not otherwise stated. *Escherichia coli* BL21(DE3) strain was purchased from New England Biolabs. Media and buffers were prepared in loco.

Methods

^{1}H NMR spectra were recorded on a Bruker AVA300 spectrometer (300 MHz) at 298 K in deuterated solvents.

For Gel Permeation Chromatography (GPC) measurements, samples were dried, resuspended in the GPC solvent, and shaken for several hours before being filtered with a 0.45 um PTFE filter. The measurements were carried out on Agilent 1260 Infinity instruments equipped with refractive index (RI) and UV-vis detectors. PSS Suprema columns were used in aqueous solvent (0.1 M $NaNO_3$ in ultrapure water) at 25 °C, PSS Gram Linear columns were used for DMF (+ 1 g/L LiBr) at 50 °C, and SDV linear-M columns were used for THF at 25 °C. Samples were run at a flow rate of 1 mL/min. PSS ReadyCal-kit Pullulan, PSS ReadyCal-kit PEO/PEG, and PSS ReadyCal-kit PS were used for calibrating the instruments. Number average molar mass (M_n) and dispersity (Đ) values were determined with the PSS WinGPC software. Fluorescence spectra were recorded on a HORIBA Fluoromax+, using 1 cm path length cuvettes, and analyzed using Origin software. Flow cytometry analysis was carried out on a Beckman Coulter CytoFLEX, and confocal images were taken on a Leica DMi8 inverted microscope. LEICA software was used for the image acquisition. A BMG Labtech CLARIOstar Microplate Reader was used for cellular assays and cytotoxicity assays. Experiments in 96-well plates under anoxic conditions were carried out in a glove box MBraun UNIlab filled with nitrogen. SDS-PAGE gels were run with a Bio-Rad protein electrophoresis equipment on a 12.5% polyacrylamide gel. Cells were lysed on ice using a Fisherbrand™ Model 505 Sonic Dismembrator with a maximum amplitude of 37%, 10 bursts of 15 seconds every 30 seconds.

Cell transformation and protein expression

Competent *E. coli* BL21(DE3) cells were transformed with a pET-21a vector carrying a wild-type (WT) sperm whale myoglobin gene or an empty pET-47b vector carrying only the antibiotic resistance by standard heat-shock protocol. The obtained colonies were sequenced before preparing the glycerol stock. Cells were then grown, harvested, and lysed as described below. Induction of the recombinant protein was carried out in Terrific Broth (TB). 1 mM IPTG and 3 mM 5-aminolevulinic acid (ALA) were added to the culture when the OD_{600} reached approximately 0.8, and the induction was performed overnight at 37°C.

Cell culture and lysate

Precultures of *E. coli* BL21(DE3) were grown overnight at 37 °C in 10 mL of fresh Luria-Bertani medium (LB) supplemented with proper antibiotics, starting from glycerol stocks. The precultures were then used to inoculate fresh LB media (1 mL for every 100 mL of fresh media) and grown at 37 °C under constant shaking. Overnight cultures were used to produce the cellular lysate by resuspending the pellet of 1 L culture in 14 mL phosphate-buffered saline (PBS) before lysing the cells by ultrasonication. 5 hours cultures were used for the intracellular polymerizations.

Synthesis of N-isopropyl 2-bromopropionamide (NIPBPA)

A round-bottom flask under protective argon atmosphere was placed in an ice bath and loaded with 1.53 g (10 mmol) of 2-bromopropanoic acid. A mixture of 30.0 mL dimethyl sulfoxide (DMSO), 0.650 g propane-2-amine (11 mmol), 3.54 g triethylamine (35 mmol), 4.55 g hexafluorophosphate benzotriazole tetramethyl uronium (HBTU; 12 mmol) and 4.73 g hydroxybenzotriazole (HOBt; 35 mmol) was slowly added to the flask under stirring. After the addition ended, the flask was removed from the ice bath and left to warm up to 25 °C for 24 h. The reaction was resuspended in ethyl acetate, then washed with water and brine, dried over $MgSO_4$, and concentrated *in vacuo*, before being purified by flash chromatography (gradient of ethyla cetate / cyclohexane) using a Selekt automated flash chromatography system (Biotage) and PuriFlash columns (Si-HP 25g). The solvent of the product-containing fractions was evaporated, and the residual oil triturated with trichloromethane. Slow evaporation of the trichloromethane allowed the formation of crystalline needles of pure N-isopropyl 2-bromopropionamide (700 mg, 55% yield).

^{1}H NMR ($CDCl_3$, 300 MHz): δ 6.26 (NH (D), br, 1H), 4.40 (CHBr (B), q, *J* = 6.9 Hz, 1H), 4.05 ($CHMe_2$ (E), qd, *J* = 13.6, 6.7 Hz, 1H), 1.88 (CH3 (C), d, *J* = 7.1 Hz, 3H), 1.20 (CH3 (F-G), d, *J* = 6.5 Hz, 6H).

^{13}C NMR ($CDCl_3$, 75 MHz): δ 168.5 (A), 45.6 (B), 42.3(E), 23.3 (C), 22.4, 224 (F-G).

MS (ESI-TOF) m/z: [M + Na]+ Calc for C6H12NOBr 194.01, 196.01; Found 194.018 196.012.

General polymerization in aqueous solution

Liquid monomers containing inhibitors were passed through a plug of basic alumina, while solid monomers were used immediately. Three solutions were prepared and degassed by bubbling argon through them for 1 h at room temperature. The monomer was dissolved in phosphate buffer with added sodium bromide (PB-Br; pH 7.4; 100 mM NaBr). Sodium ascorbate (NaAsc) and the initiator were mixed in PBS-Br and DMSO as a cosolvent, and commercial myoglobin was solubilized in PBS-Br in a Schlenk vial (10 mL). Typically, the cosolvent concentration was kept below 12% of the total final volume, and the initiator concentration was kept between 1 mM and 30 mM, depending on the initiator and the conditions. Finally, a vial with PBS-Br was degassed for 1 h under argon before adding sodium dithionite (NaDT). Some microliters of fresh NaDT solution were then immediately transferred to the Schlenk flask to reduce the iron of the protein to Fe^{2+}. Then, the two solutions containing the initiator and the monomer were transferred to the Schlenk flask to start the reaction. For all experiments, the molar ratio was maintained unchanged as follows: initiator(1): monomer(50): NaAsc(1): myoglobin(0.005): NaDT(0.005).

Polymerization in cellular lysate

The experiment was repeated following the same procedure used for the polymerization in PB-Br, but the cosolvent was reduced to 1% to 2% of the total volume.

Polymerization in cells

200 mL of *E. coli* culture grown in LB was kept under argon and mild shaking using an orbital shaker mixer for 1 h in a 500 mL Duran bottle closed with a rubber septum. At the same time, a solution of the initiator in 4 mL of LB and 1 mL of DMSO and a solution of monomer(s) in 40 mL of LB were degassed by gently bubbling with argon. The initiator and the monomer were then transferred to the bottle under argon, and the bottle was transferred to a shaking incubator. The reaction was carried on at 37 °C or 28 °C for 2 or 4 hours under constant shaking, with a final monomer

concentration of 50 mM (AAm, NAM, NIPAm), 20 mM (HEMA, HPMA) or 0.02 mM (FOM) and a final initiator concentration of 1 mM.
Control experiments were performed following the same protocol. In these, the initiator was replaced by the same volume of LB or PBS.
To test the polymerization, the cells were pelleted, washed three times in 50 mL PBS (5000 g, 10 minutes, 4 °C), and frozen at -20 °C overnight. The frozen pellet was then thawed, resuspended in 2 mL ultrapure water, and sonicated on ice. The polymer was extracted from the lysate according to its physiochemical characteristics, as described below.

Polymer extraction from the cells

PAAm

The cell debris were removed by centrifugation (16000 g, 3 minutes), and the supernatant was dried and resuspended in 650 µL of D_2O. After removing the insoluble residues, the sample was analyzed using NMR spectroscopy and GPC.

PNIPAm

The cell debris was removed by centrifugation (16000 g, 3 minutes), and the supernatant was heated to 50 °C for half an hour. After that, the solution was centrifuged again (16000 g, 2 minutes, 40 °C), and the pellet was resuspended in 650 µL of icy D_2O. After removing the insoluble residues, the sample was analyzed using NMR spectroscopy and GPC.

PNAM

The cell debris was removed by centrifugation (16000 g, 3 minutes), and the supernatant was dried. Cell residues were resuspended in 2 mL of DMF. The insoluble residues were then removed by centrifugation, and the obtained solution was dried again. Finally, the resulting dried compounds were solubilized in 650 µL of D_2O and analyzed by NMR spectroscopy and GPC.

PHEMA and PHPMA

The cellular lysate was centrifuged (16000 g, 3 minutes), the supernatant discharged, and the pellet resuspended in 2 mL of DMSO. Anything not solubilized was then removed by centrifugation, and the supernatant dried. The dry residue was then dissolved in 650 µl d_6-DMSO and analyzed by NMR spectroscopy and GPC.

Free radical copolymerization of AAm and FOM

The free radical polymerization of acrylamide and FOM was carried out in PBS using sodium thiosulfate and potassium persulfate. 0.0632 g sodium thiosulfate, 0.0026 g FOM, and 0.1402 g AAm were dissolved in 1.9 mL PBS. A solution of 0.3243 g potassium persulfate in 1 mL PBS was prepared separately. The two solutions were degassed for 30 minutes, and then 210 µL of potassium persulfate solution was transferred into the reaction mixture to start the reaction.

Cell viability

After the reaction, a 500 µL sample was taken from the reaction bottle or the control tubes. The cells were pelleted (10000 g, 1 min) and washed two times in fresh PBS before resuspending them in 500 µL of fresh sterile LB supplemented with the proper antibiotic. Each sample was then diluted following a standard serial dilution protocol in sterile LB, and 50 µL of the chosen dilution was

plated on an LB-agar plate and incubated overnight at 37 °C or 24 h at 30 °C. The colonies were then manually counted, and the number of CFU/mL was calculated according to the following equation:

CFU/ml = (no. of colonies x dilution factor) / volume of culture plate

The percentage of the survived cells of each experiment was then calculated according to this equation:

Survival % = [log(treated) / log(untreated)] x 100 %

Membrane integrity assay

A maximum of twelve different mixes were screened in parallel, following the same protocol used for the polymerization in cells but in a final volume of 1.225 mL. Each mix was prepared in six different wells from previously degassed solutions of monomers in LB, initiators in LB with 5% DMSO, LB with 5% DMSO, or pure LB. An untreated control was always prepared by mixing 1 mL of cell culture with 100 µL pure LB and 125 µL LB with 5% DMSO. The experiments were conducted in a 96-well deep plate under constant shaking at 37 °C or 28 °C inside a glove box filled with nitrogen. After 2 or 4 hours of incubation, the 96-well plate was removed from the glove box, and the cells were washed 2 times in PBS. Three of six replicates were resuspended in PBS, while the rest were quickly resuspended in a 50:50 v/v isopropanol-PBS solution to produce 100% permeable cells. The cells were then washed in PBS and resuspended in the staining buffer (PBS containing 1 µL/mL of 2 mg/mL propidium iodide stock solution in DMSO). After 5 minutes of incubation in the dark, the fluorescent intensity at 630 nm (excitation: 520 nm) was recorded in a black F-bottom 96-well plate.

The percentage of permeable cells was calculated as follows:

((T-B)/(C-B)) x 100 %

where T is the tested condition, C is the control treated with isopropanol, and B is the staining solution. The untreated control was then removed from the value of the treated cells to give only the contribution of the tested monomer/initiator combinations.

Cytotoxicity assay

Cells were grown in LB until an OD_{600} between 0.6 and 1 and subsequentially diluted. Stock solutions were prepared in LB or pure DMSO. Dilutions of each stock were prepared according to the final concentration needed.

10 µL of monomer in LB solution at different concentrations were added to 90 µL of cells in LB, or 1 µL of initiator in DMSO was added to 99 µL of cells in LB. Bacteria were tested starting from an OD ~ 0.0015 at time zero. The plate was then incubated for 6 hours at 37 °C under shaking in aerobic conditions. The growth was stopped by cooling the plate on ice, and the OD_{600} was recorded in a plate reader. The blank value from pure LB, or LB with 1% DMSO, was subtracted from each value, and the growth inhibition was then calculated for each concentration as follows:

((Control - Treated) / (control)) x 100 %

Control cells were prepared for each molecule tested by adding simple LB and/or DMSO to the cells. The obtained values were plotted against the tested molecule concentration, and the IC50 was calculated, fitting the data with a straight line.

$$IC50 = (0.5 - b)/a$$

where a is the slope and b is the y-intercept of the linear equation.

Dead/alive cell staining for microscopy

After reaction or treatments, cells were accurately washed in PBS and stained in a solution of 150 µL/mL of fluorescein diacetate (FDA) in PBS for 30 minutes at 37 °C. Cells were then washed and resuspended in a solution of 2 µg/mL propidium iodide (PI) in PBS and incubated for 5 minutes in the dark before being washed once more and resuspended in PBS to be imaged.

Supplementary Data

Table S1. Influence of the temperature on myoglobin-catalyzed polymerization in cellular lysate.

	Room temperature			37 °C		
	conver-sion	M_n (g/mol)	Đ	conversion	M_n (g/mol)	Đ
Acrylamide	52%	4.22 x 10^4	1.82	80%	3.90 x 10^4	1.66
NAM	48%	1.87 x 10^4	1.95	85%	1.15 x 10^4	1.72

The reactions were performed with 30 mM EBPA as initiator, with a monomer-to-initiator ratio of 50:1, a myoglobin concentration of 0.15 mM, and a myoglobin-to-reducing agent ratio of 1:1 Myo:NaDT, and 1:200 Myo:NaAsc. Polyacrylamide was analyzed on an aqueous GPC using 0.1 mol/L $NaNO_3$ aqueous solution as eluent and pullulan standards calibration at 25°C. PNAM was analyzed on a DMF (1 g/L LiBr) GPC using PEO-PEG standards calibration at 50 °C.

Table S2. Screening of the reaction conditions.

PBS

Acrylamide	EBPA	Commercial Mb	NaAsc	NaBr	Reaction time	Conversion	Đ	M_n (g/mol)
✓	✓	✓	✗	✓	4 hours	0%	-	-
✓	✓	✗	✓	✓	4 hours	10%	1.74	8.15×10^4
✓	✓	✗	✗	✓	4 hours	0%	-	-
✓	✓	✓	✓	✓	4 hours	65%	1.58	4.62×10^4

Cellular lysate from *E. coli* BL21(DE3)

Acrylamide	EBPA	Commercial Mb	NaAsc	NaBr	Reaction time	Conversion	Đ	M_n (g/mol)
✓	✓	✓	✗	✓	1 hour	80%	1.66	4.90×10^5
✓	✓	✗	✓	✓	4 hours	25%	1.63	7.60×10^4
✓	✓	✗	✗	✓	1 hour	90%	1.38	1.18×10^6
✓	✓	✓	✓	✓	1 hour<br>4 hours	35%<br>80%	not measured<br>1.68	not measured<br>3.11×10^4
✓	✓	✗	✗	✗	1 hour	90%	1.26	1.24×10^6
✓	✗	✗	✗	✓	4 hours	3%	-	-

Cellular lysate from *E. coli* BL21(DE3) - induced expression of myoglobin

Acrylamide	EBPA	Expressed Mb	NaAsc	NaBr	Reaction time	Conversion	Đ	M_n (g/mol)
✓	✓	WT	✗	✗	1 hour	90%	1.71	1.76×10^5
✓	✓	WT	✓	✗	4 hours	85%	1.74	2.71×10^4
✓	✗	WT	✓	✗	4 hours	0%	-	-

All reactions were performed at 37°C in anoxic condition with a monomer-to-initiator ratio of 50:1 and an initiator concentration of 30 mM. The conversion was monitored by ^{1}H NMR spectroscopy. Polyacrylamide was analyzed on an aqueous GPC using 0.1 mol/L $NaNO_3$ aqueous solution as eluent and pullulan standards calibration at 25°C.

Table S3. Monomer/initiator screening with myoglobin as catalyst.

Initiator	Monomer	Conversion	Condition	Temperature
EBPA	Acrylamide	70% / 90%	PBS / Lysate	RT / 37 °C
	NAM	60% / 90%	PBS / Lysate	RT / 37 °C
	NIPAm	60% / 90%	PBS / Lysate	25 °C
	HPMAm	-	-	-
	HPMA	>60%	PBS	RT
	NaSS	-	PBS / Lysate	RT / 37 °C
	2MEA	70%	Lysate	37 °C
HEBIB	Acrylamide	5% / 10%	PBS / Lysate	RT / 37 °C
	NAM	>90%	Lysate	37 °C
	NIPAm	>90%	Lysate	28 °C / 37 °C
BPAA	NIPAm	80%	Lysate	28 °C
	NAM	65%	Lysate	37 °C
NIPBPA	NIPAm	60%	Lysate	28 °C
MBPA	Acrylamide	60%	Lysate	37 °C
	NaSS	90%	Lysate	37 °C
BPN	Acrylamide	-	PBS	-
	HPMAm	-	PBS	-

Reactions were performed in cellular lysate or PBS with 0.15 mM myoglobin at different temperatures in anoxic conditions with a maximal monomer-to-initiator ratio of 50:1 and an initiator concentration between 1 mM and 30 mM. The success of the polymerization was determined by ^{1}H NMR spectroscopy.

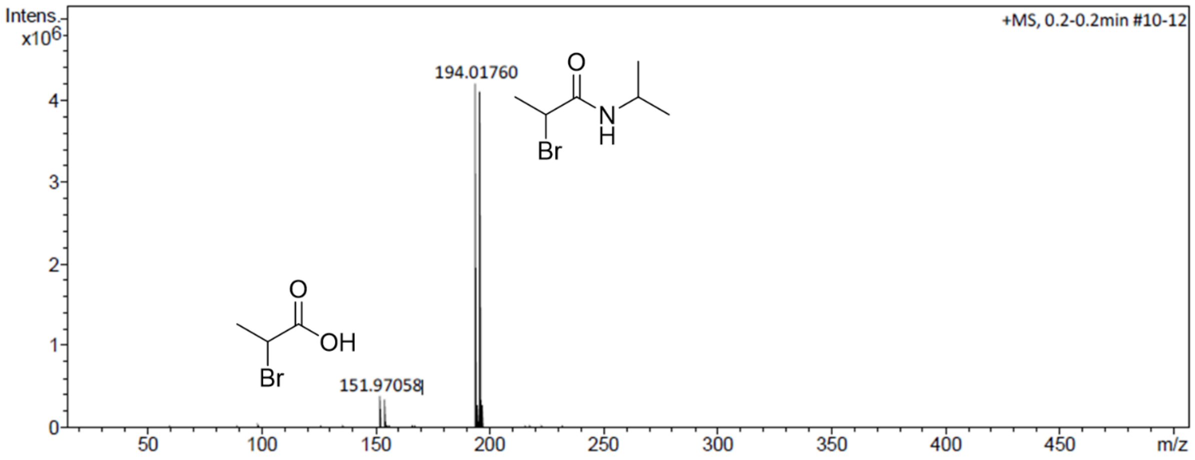

Figure S1. Mass spectrum of the synthesized initiator NIPBPA.

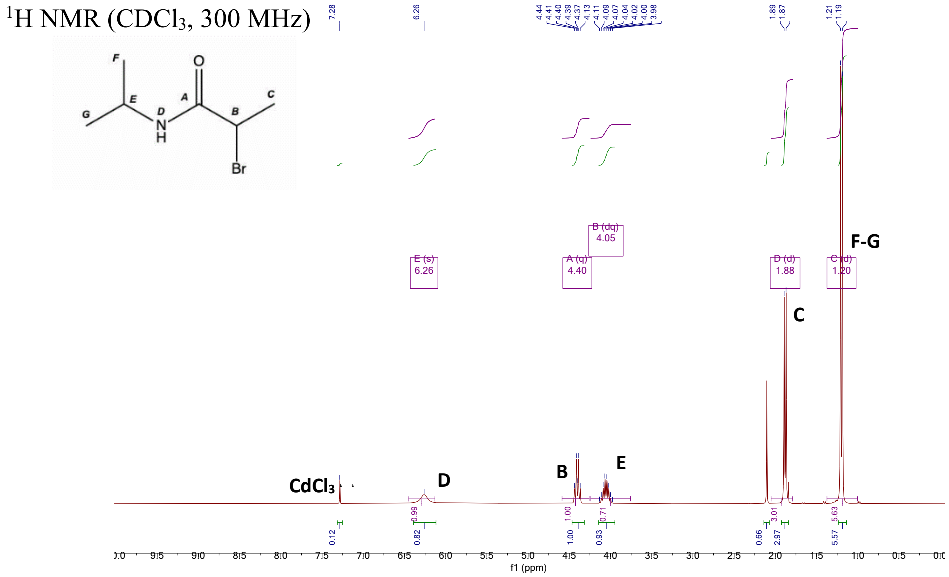

Figure S2. ^{1}H NMR spectrum of the synthesized initiator NIPBPA.

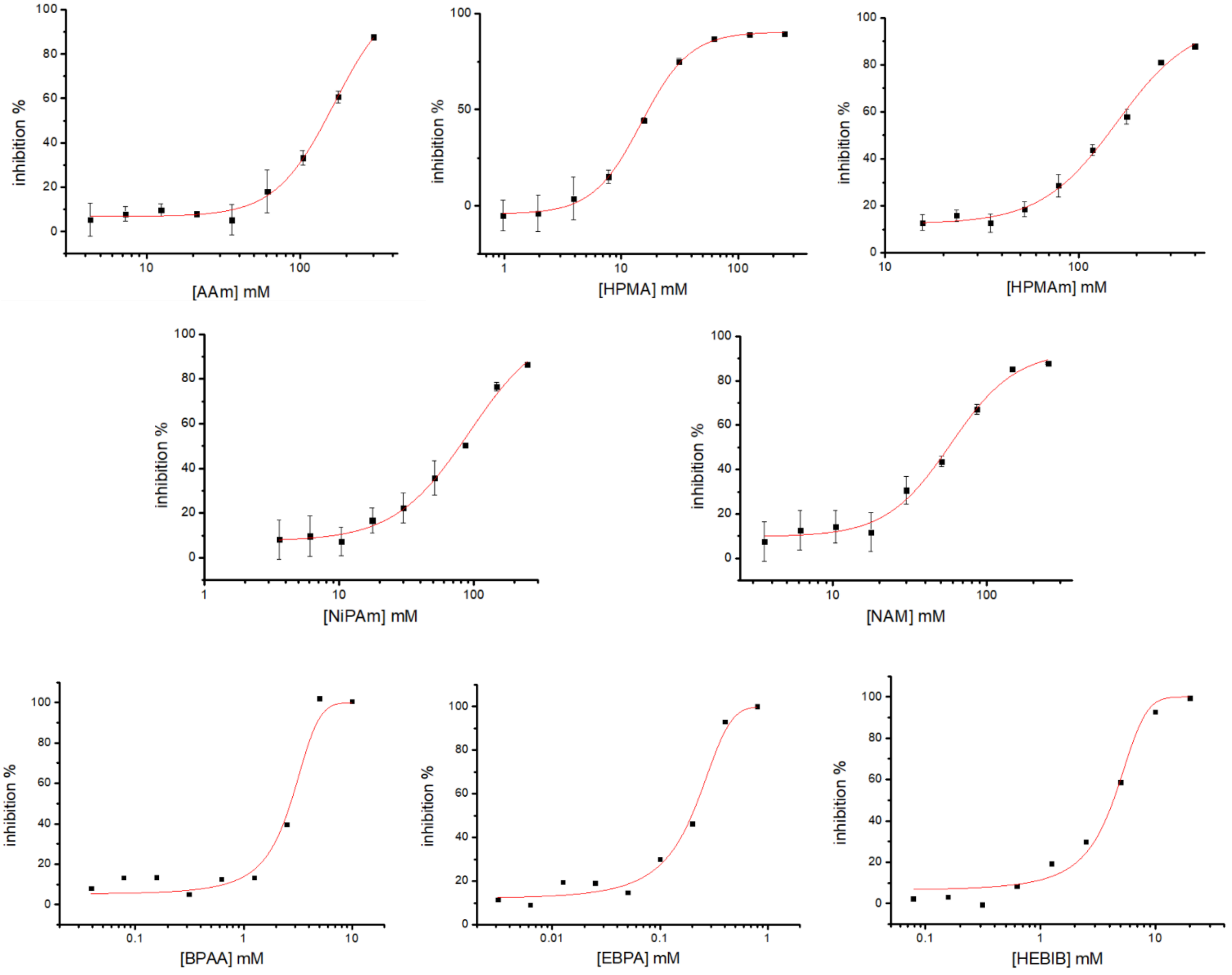

Compound name and abbreviation	Concentration range (mM)	IC_{50} mM
Acrylamide (AAm)	4.3 – 300	166.3
2-Hydroxypropyl methacrylate (HPMA)	1 - 250	14.9
N-(2-hydroxypropyl) methacrylamide (HPMAm)	15.6 - 400	157.7
N-isopropylacrylamide (NIPAm)	3.57 – 250	92.5
N-acryloyl morpholine (NAM)	3.57 - 250	58.3
α-Bromophenylacetic acid (BPAA)	0.04 – 10	2.70
Ethyl α-bromophenylacetate (EBPA)	0.003 - 0.8	0.16
2-Hydroxyethyl 2-bromoisobutyrate (HEBIB)	0.08 - 20	4.33

Figure S3. Inhibition curves, concentration range used for IC_{50} determination (n = 3), and calculated IC_{50} values.

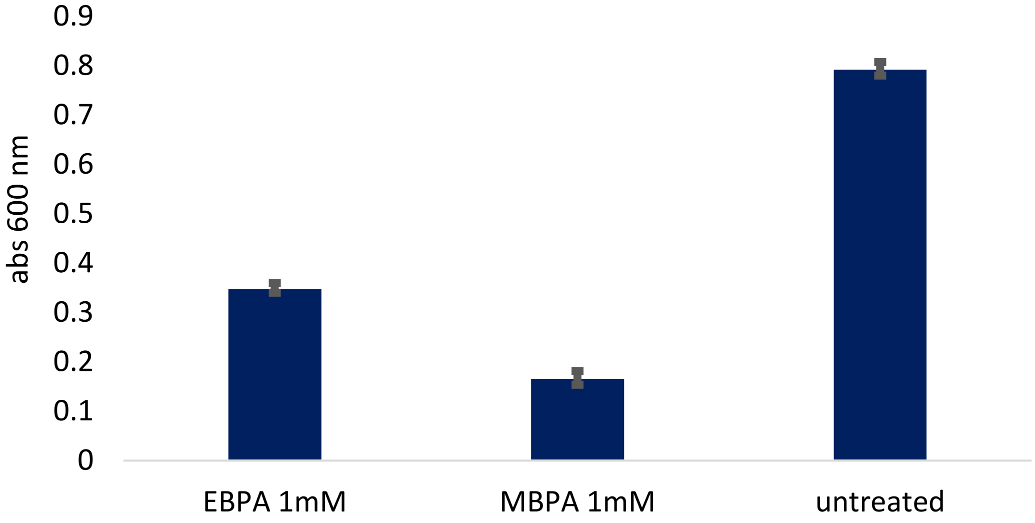

Figure S4. Comparison of the toxicity of EBPA and MBPA. 99 µl of cell suspension at an OD_{600} of 1 were treated with 1 µL of DMSO solution of 0.1 M EBPA or 0.1 M MBPA. Pure DMSO was used for the untreated cells. After 6 hours of incubation at 37°C in aerobic conditions, cells were diluted, and the OD_{600} was measured again. The treatment with EBPA and MBPA resulted in 56% and 79% fewer cells compared to the control. (EBPA ± 0.01; MBPA ± 0.014; untreated ± 0.014; (n = 3)).

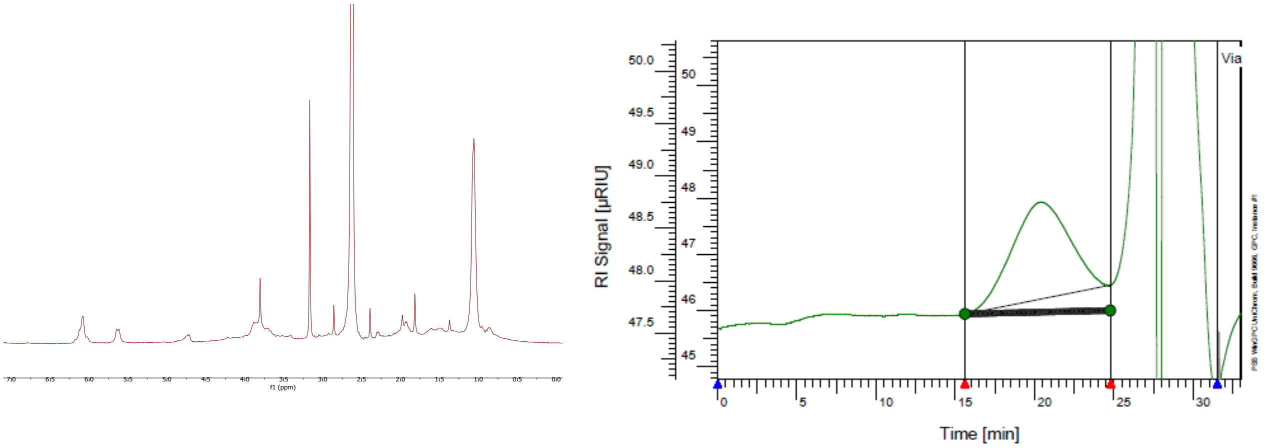

Figure S5. ^{1}H NMR spectrum and GPC trace of PNIPAm synthesized in cellular lysate from 50 mM NIPAm and 1 mM EBPA.

Table S4. Molecular weight and dispersity of different polymers extracted from the cells.

HEBIB

Monomer	M_n (g/mol)	Đ
HPMA	2.44 x 10^4	1.87
HEMA	7.37 x 10^4	1.67
NAM	1.96 x 10^4	1.74
NIPAm, 37 °C	5.85 x 10^4	2.07
NIPAm, 28 °C	2.43 x 10^4	1.84

EBPA

Monomer	M_n (g/mol)	Đ
AAm	> 1.00 x 10^6	-
HEMA	7.73 x 10^4	1.55
NIPAm, 28 °C	1.06 x 10^4	1.57

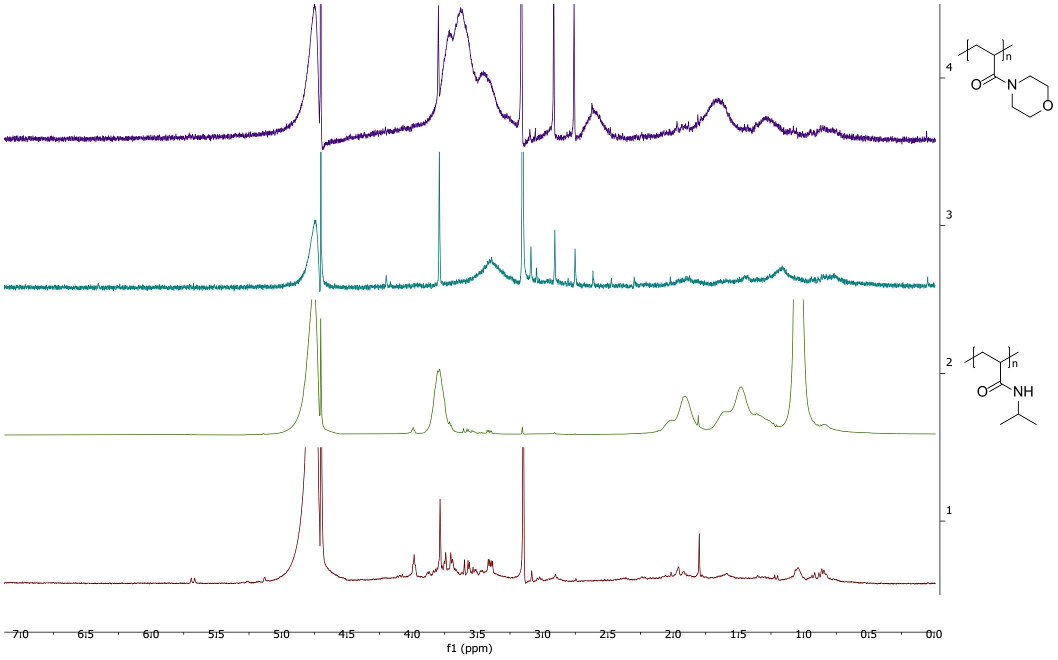

Figure S6. ^{1}H-NMR spectra comparison of the extracted cellular content after intracellular polymerization using HEBIB (2, 4) or BPAA (1, 3) as initiator, and NAM (3, 4) or NIPAm (1, 2) as monomers. No detectable polymer was extracted from the cells when BPAA was used as the initiator.

Table S5. Comparison of polymers synthesized under different conditions with HEBIB as initiator.

monomer	condition	Đ	M_n (g/mol)
NAM	Cellular lysate	2.89	1.28×10^5
NAM	Cells	1.73	1.96×10^4
NIPAm	Cellular lysate	3.56	1.68×10^5
NIPAm	Cells	1.93	5.15×10^4

All reactions were performed at a final monomer/initiator ratio of 50:1, without any reducing agent or added catalyst. 30 mM HEBIB was used for the reaction in cellular lysate, while 1 mM HEBIB was used for the reaction in cells. For the reaction in cellular lysate, 200 µL of the reaction mixture were taken for NMR and GPC analysis. The cells were polymerized, and the polymer was extracted from the cells as described after 4 h.

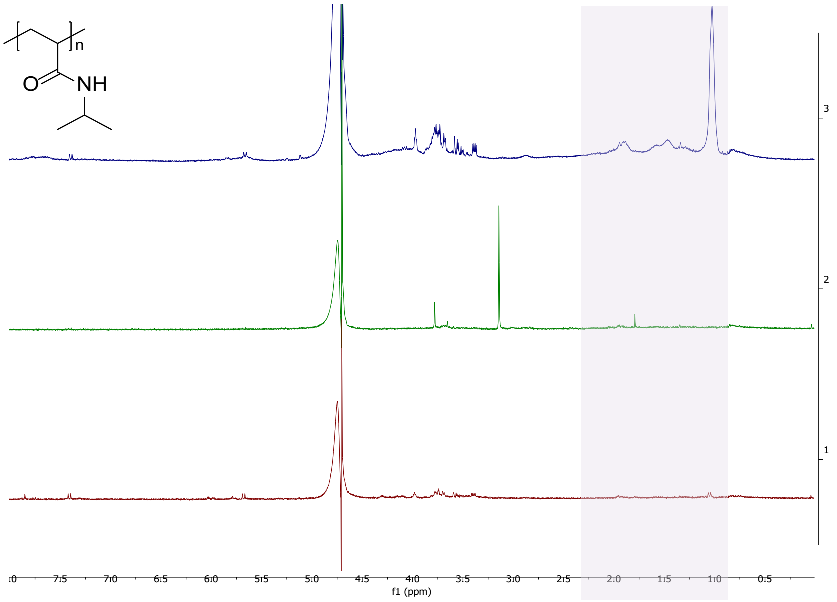

Figure S7. ^{1}H NMR spectra of three experiments to demonstrate that initiator, monomer, and anoxic conditions are required for the intracellular polymerizations. (1) Cells incubated with 50 mM NIPAm and 1 mM HEBIB under aerobic conditions, (2) cells fed with only 50 mM NIPAm in anaerobic conditions, and (3) cells fed with 50 mM NIPAm and 1 mM HEBIB in anaerobic conditions. The spectral range highlighted is where the signals of protons on the polymer backbone are. The spectra show that PNIPAm was only extracted from cells fed with monomer and initiator under anoxic conditions.

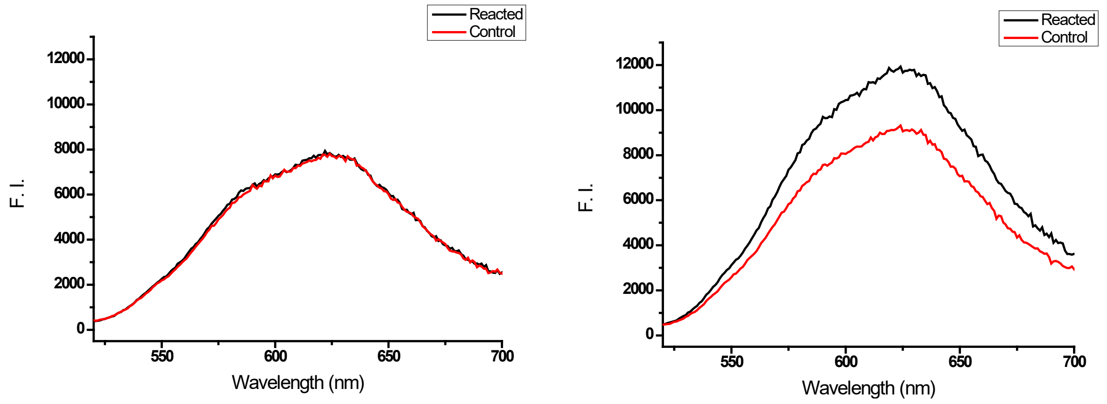

Figure S8. Fluorescence emission spectra of reacted and control cells in a 10 μg/mL solution of Nile Red in PBS before and after sonication. Cells were polymerized for 2 hours at 37 °C under anoxic conditions with 20 mM HEMA and 1 mM HEBIB. The control was prepared under the same condition, omitting the initiator. After the reaction, cells were washed three times in PBS and incubated with the dye for 30 minutes in the dark before spectra acquisition. The cells were then lysed by sonication, and the spectra were acquired again. Excitation: 488 nm; emission 520 – 700 nm; gain 1400; focal high 9.8 mm.

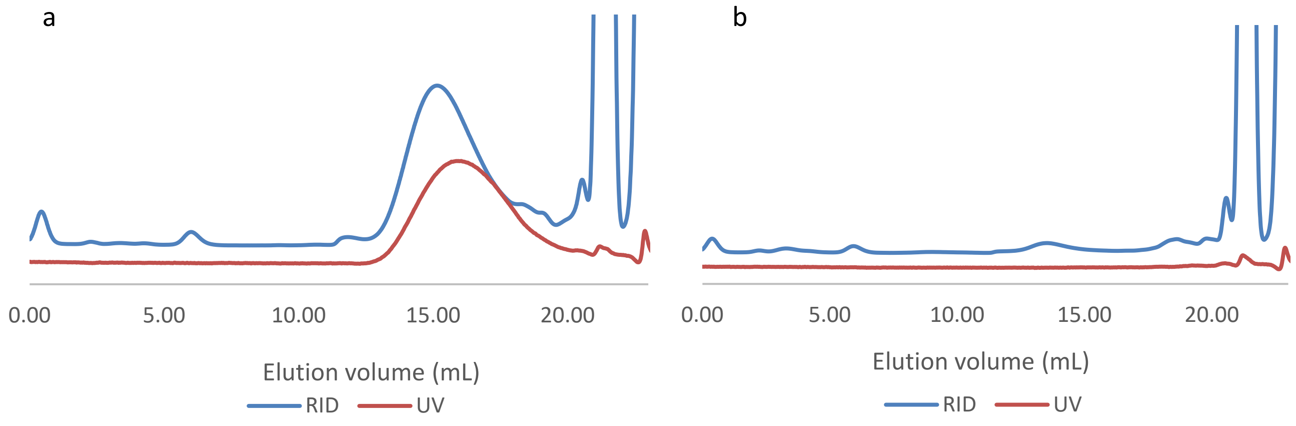

Figure S9. (a) GPC traces of the reaction mixture of the copolymerization of AAm and FOM with the initiator EBPA. (b) GPC traces of a control reaction without the initiator. Reaction conditions: 0.25 M AAm, 8 x 10^{-4} M FOM, 8 x 10^{-3} M EBPA in cellular lysate from *E. coli* BL21(DE3), reaction time: 1 hour at 37 °C. The UV-vis detector was set to 488 nm.

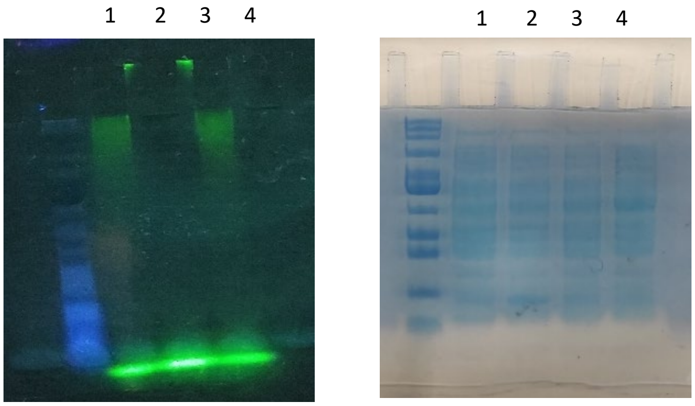

Figure S10. SDS-PAGE gel of P(AAm-*co*-FOM) synthesized in cellular lysate. Two different reaction mixtures (1 and 3) of 0.25 M AAm, 0.8 mM FOM, 8 mM EBPA in cellular lysate from *E. coli* BL21(DE3), a control reaction without initiator (2) and the cellular lysate itself (4). Left: gel illuminated with a UV lamp at 366 nm. Right: gel stained with Coomassie-blue to highlight the protein content of the samples.

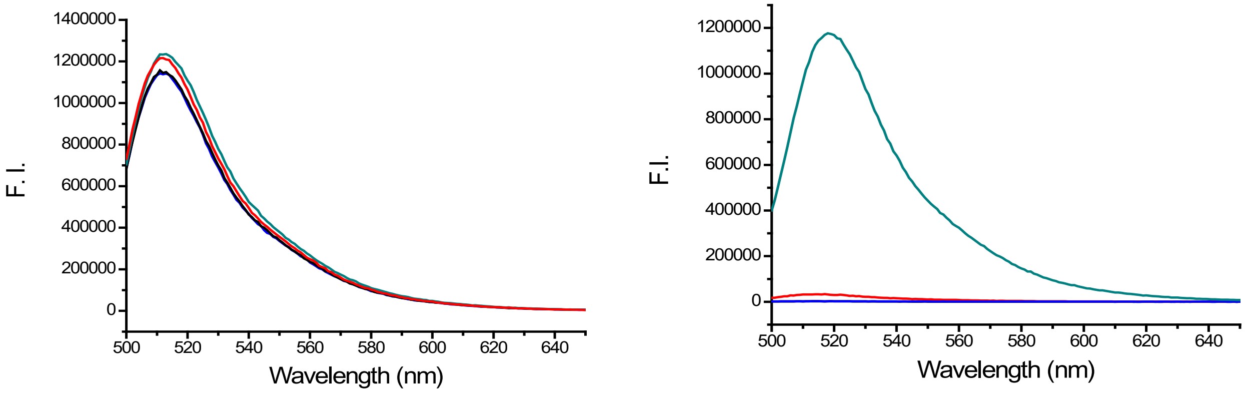

Figure S11. Emission spectra of a reaction of AAm, FOM, and EBPA in cellular lysate at time zero (black line) and after 2 hours of reaction (green line); and the same reaction without EBPA at time zero (blue line) and after 2 hours of reaction (red line), before (left) and after (right) purification with a PD10 column to remove unreacted fluorescent monomer. Only the reaction mixture containing the initiator resulted in fluorescence after purification, confirming the presence of the fluorescent P(AAm-*co*-FOM) copolymer. Moreover, the spectra allow to conclude that the fluorescence intensity of FOM does not change during the polymerization, as can be seen by the similar fluorescence intensity of the non-purified samples taken at the beginning and the end of the reaction. Excitation: 480 nm, slits 1/1.

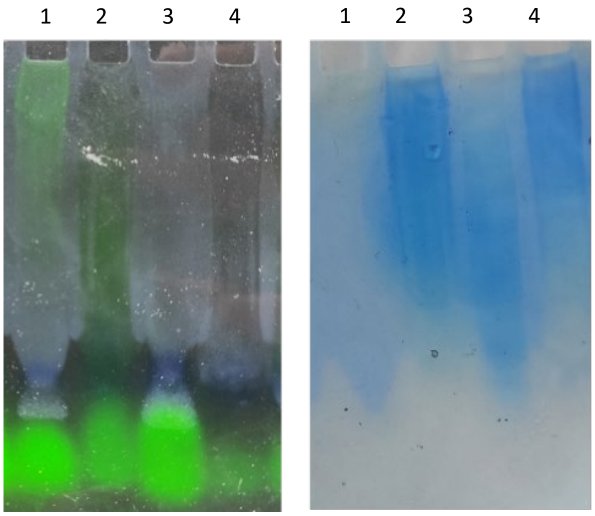

Figure S12. SDS-PAGE gel of P(NIPAm-*co*-FOM). Free radical polymerization of AAm and FOM (1); poly(NIPAm-*co*-FOM) synthesized in cellular lysate (0.44 M NIPAm, 1.5 mM FOM, 15 mM HEBIB) (2); its control reaction without initiator (3); and PNIPAm synthesized in cellular lysate and then mixed with FOM in aerobic conditions (4). Left: gel under ambient light. Right: gel stained with Coomassie-blue to highlight the presence of the polymer and proteins in the samples.

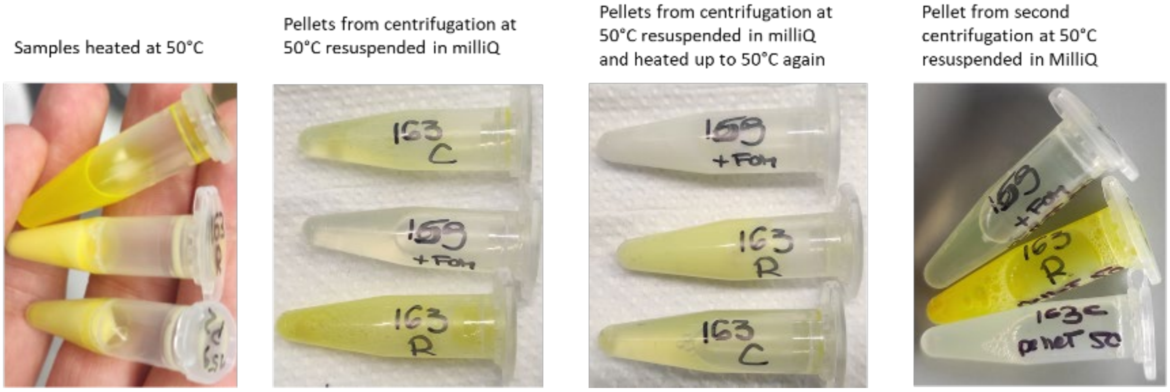

Figure S13. Sequential precipitation and solubilization of the temperature-responsive P(NIPAm-*co*-FOM) produced in cellular lysate to verify the incorporation of the fluorescent monomer in the polymer chain. Photographs of samples from the copolymerization in cellular lysate (labeled as 163R), its control experiment without initiator (163C), and from a sample in which FOM was added to PNIPAm that had been previously produced in cellular lysate (159 + FOM).

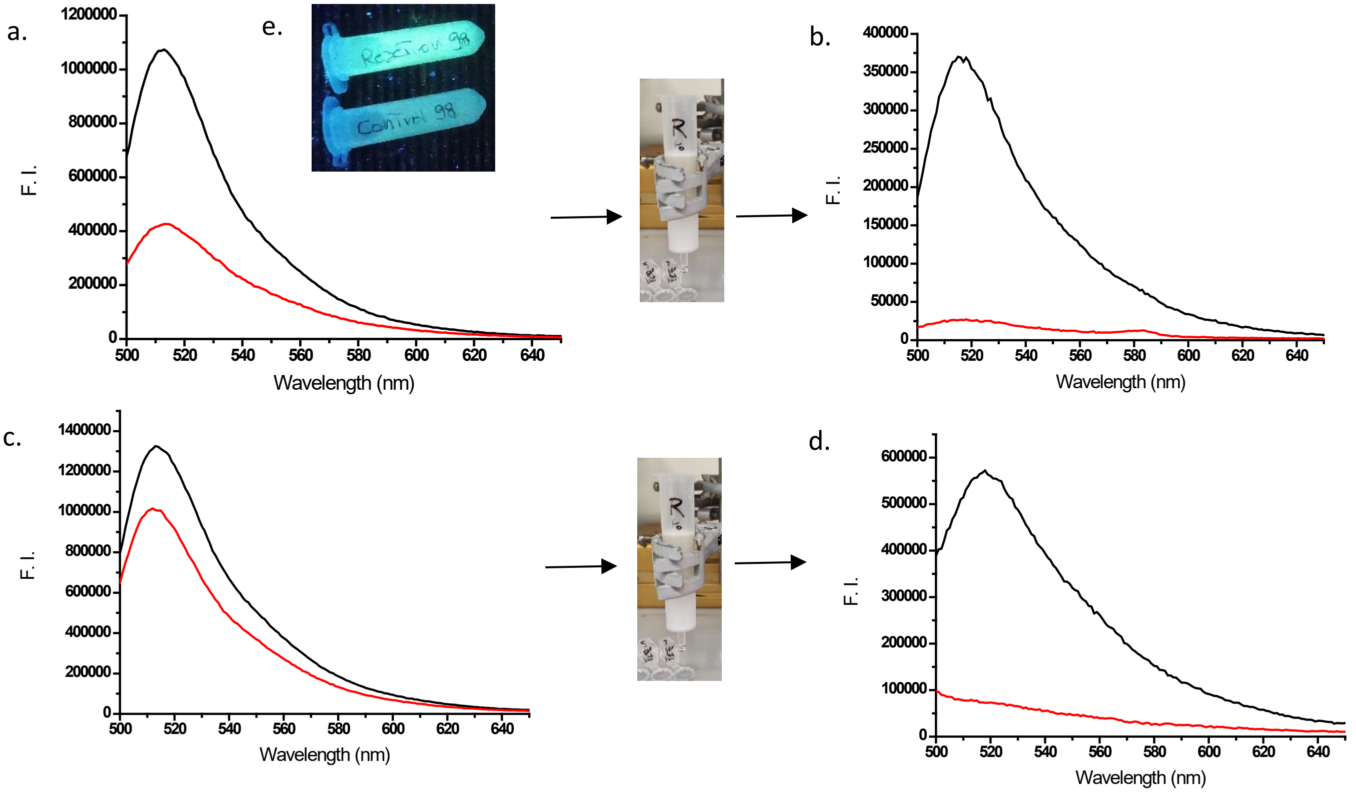

Figure S14. Fluorescence emission spectra of cell-free extract from polymerized (black line) and control (red line) cells, before (left) and after (right) Sephadex column. (a - b) Cells were treated with 50 mM AAm, 0.02 mM FOM, with or without 1 mM EBPA under anoxic conditions at 37 °C for 4 h. (c – d) Cells were treated with 50 mM NIPAm, 0.02 mM FOM, with or without 1 mM HEBIB under anoxic conditions at 28 °C for 4 h. The cells were then washed and lysed, and the supernatant was analyzed in fluorescence spectroscopy, showing higher fluorescence of the extract of polymerized cell. The sample was then purified with a PD-10 column to remove the free monomers and small molecules, and the resulting solution was analyzed again, revealing a greater difference in fluorescence intensity between the extracts of reacted and control cells. Ex: 480 nm, em: 500-650 nm, slit 2/2. (e.) Photographs of cellular extract after intracellular polymerization of acrylamide and FOM.

Table S6. Growth of *E. coli* BL21(DE3) cells in which P(NIPAm-*co*-FOM) was synthesized and of control cells.

	OD_{600} Reacted cells	OD_{600} Control cells
After reaction	1.0	1.1
1 hour of growth	0.3	0.4
2 hours of growth	0.9	1
3 hours of growth	1.4	1.5

Cells were treated with 50 mM NIPAm, 0.05 mM FOM, with and without 1 mM HEBIB and let to react in anoxic environment for 4 h at 28 °C. The OD600 was measured immediately after the reaction, and the cells were diluted 1 to 10 in fresh LB and were let grown at 37 °C in a 2 mL Eppendorf tube. The OD600 was measured hourly.

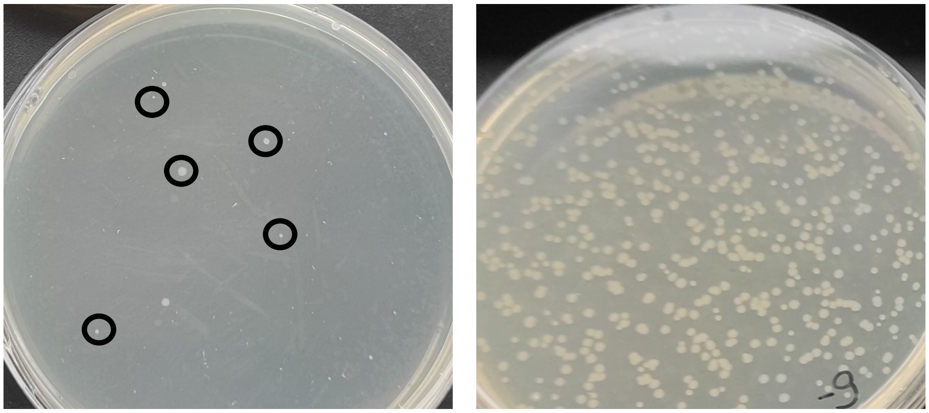

Figure S15. Cytotoxicity of EBPA. Left: Photography of an agar plate of *E. coli* BL21(DE3) treated with EBPA. Right: Photography of an agar plate of *E. coli* BL21(DE3) control sample.
A 5-hour culture of *E. coli* in LB was kept under argon for 1 hour at room temperature, treated with a degassed solution of EBPA in DMSO (final concentration 1 mM EBPA/0.4% DMSO) or LB/0.4% DMSO as control and kept under argon for further 4 hours at 37°C. Then, the cells were diluted and plated onto an agar plate and incubated at 37 °C overnight before the photos were taken. For the EBPA-treated sample: 50 µL of a 1 x 10^7-fold diluted cell suspension in LB was plated. For the control: 50 µL of a 1 x 10^9-fold diluted cell suspension was plated. Cells treated with EBPA show an irregular and slower growth, with colonies that differ in size and shape, as highlighted by the black circles, compared to the untreated control, revealing the cytotoxicity of the molecule.

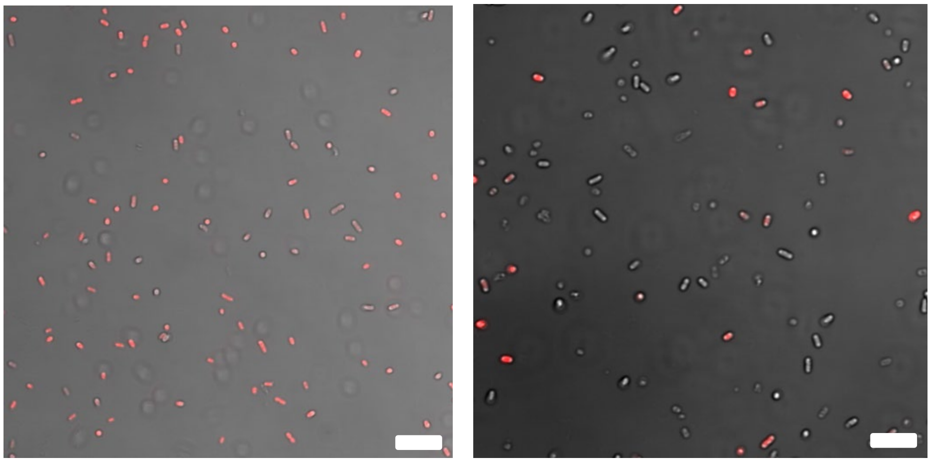

Figure S16. Confocal fluorescence microscopy images (overlay of red and transmission channels) that investigate the effect of oxygen on the cytotoxicity of EBPA. A 5-hour culture of *E. coli* BL21(DE3) in LB was treated with a solution of EBPA in DMSO under aerobic conditions and kept under argon for 2 hours at 37 °C (left), or it was kept under argon for 1 hour, treated with a degassed solution of EBPA in DMSO and kept under argon for 2 hours at 37 °C (right). Final EBPA concentration: 1 mM; final DMSO concentration: 0.4%. The cells were washed 3 times in PBS, stained with 2 μg/mL PI in PBS for 5 minutes, and washed again before being imaged. Scale bars = 10 μm.

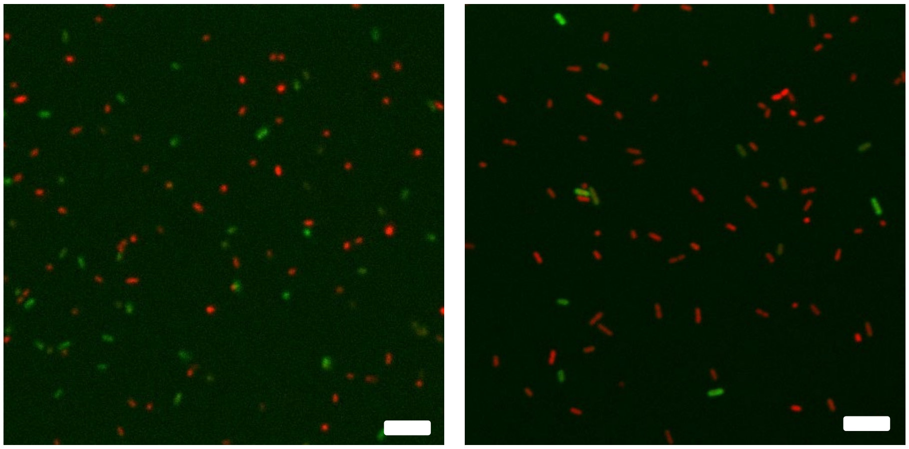

Figure S17. Confocal fluorescence microscopy images (overlay of red and green channels) reveal the toxicity of high molecular weight polyacrylamide polymerization process. *E. coli* BL21(DE3) cells during the polymerization of acrylamide at reaction times of 2 hours (left) and 4 hours (right). Cells were treated with 150 mM acrylamide and 1 mM EBPA under anoxic conditions and let react at 37 °C under shaking before being stained with FDA/PI to image membrane-damaged (red) and metabolically active (green) cells. Scale bars = 8 μm.

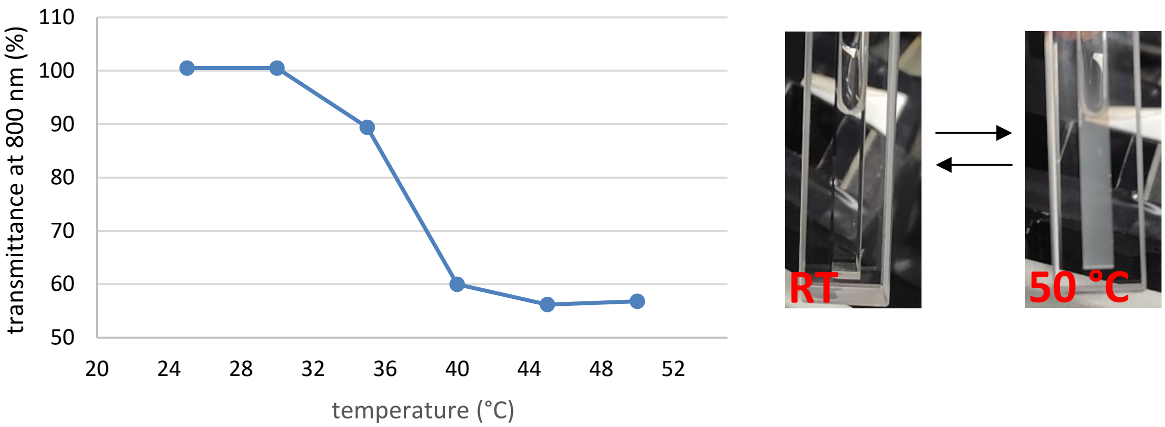

Figure S18. Determination of cloud point temperature (T_c) of PNIPAm extracted from polymerized BL21(DE3) cells and photographas of the PNIPAm solution in an optical cuvette at room temperature and its precipitate at 50 °C. PNIPAm was dissolved in a solution of 0.1 M $NaNO_3$ in ultra-pure water at room temperature. The solution was transferred into an optical quartz cuvette (1 cm path length), and its transmittance at 800 nm was measured at various temperatures with a UV-vis spectrometer featuring a thermostatted cuvette holder. The midpoint of the transition, i.e. T_c, is approx. 37 °C.
